# Increased TMEM106B levels lead to lysosomal dysfunction which affects synaptic signaling and neuronal health

**DOI:** 10.1186/s13024-025-00831-2

**Published:** 2025-04-23

**Authors:** Jolien Perneel, Miranda Lastra Osua, Sara Alidadiani, Nele Peeters, Linus De Witte, Bavo Heeman, Simona Manzella, Riet De Rycke, Mieu Brooks, Ralph B. Perkerson, Elke Calus, Wouter De Coster, Manuela Neumann, Ian R. A. Mackenzie, Debby Van Dam, Bob Asselbergh, Tommas Ellender, Xiaolai Zhou, Rosa Rademakers

**Affiliations:** 1 VIB Center for Molecular Neurology, VIB, Antwerp, Belgium; 2 Department of Biomedical Sciences, University of Antwerp, Antwerp, Belgium; 3 VIB Bioimaging Core, VIB, Ghent, Belgium; 4 Department of Biomedical Molecular Biology, Ghent University, Ghent, Belgium; 5 VIB Center for Inflammation Research, Ghent, Belgium; 6 Department of Neuroscience, Mayo Clinic, Jacksonville, FL USA; 7 Experimental Neurobiology Unit, University of Antwerp, Antwerp, Belgium; 8 Neurochemistry and Behaviour Group, University of Antwerp, Antwerp, Belgium; 9 Department of Neuropathology, University of Tübingen, Tübingen, Germany; 10 Molecular Neuropathology of Neurodegenerative Diseases, German Center for Neurodegenerative Diseases, Tübingen, Germany; 11 Department of Pathology, Vancouver Coastal Health, Vancouver, BC Canada; 12 Division of Neurology, University of British Columbia, Vancouver, BC Canada; 13 Djavad Mowafaghian Centre for Brain Health, University of British Columbia, Vancouver, Canada; 14 Department of Pathology and Laboratory Medicine, University of British Columbia, Vancouver, BC Canada; 15 Department of Neurology and Alzheimer Research Center, University of Groningen and University Medical Center Groningen, Groningen, The Netherlands; 16 State Key Laboratory of Ophthalmology, Zhongshan Ophthalmic Center, Sun Yat-Sen University, Guangdong Provincial Key Laboratory of Ophthalmology and Visual Science,, Guangzhou, 510060 China

**Keywords:** TMEM106B, Mouse model, Lysosomal dysfunction, Neuronal activity, Synaptic signaling

## Abstract

**Background:**

Genetic variation in Transmembrane protein 106B (TMEM106B) is known to influence the risk and presentation in several neurodegenerative diseases and modifies healthy aging. While evidence from human studies suggests that the risk allele is associated with higher levels of TMEM106B, the contribution of elevated levels of TMEM106B to neurodegeneration and aging has not been assessed and it remains unclear how TMEM106B modulates disease risk.

**Methods:**

To study the effect of increased TMEM106B levels, we generated Cre-inducible transgenic mice expressing human wild-type TMEM106B. We evaluated lysosomal and neuronal health using in vitro and in vivo assays including transmission electron microscopy, immunostainings, behavioral testing, electrophysiology, and bulk RNA sequencing.

**Results:**

We created the first transgenic mouse model that successfully overexpresses TMEM106B, with a 4- to 8-fold increase in TMEM106B protein levels in heterozygous (hTMEM106B(+)) and homozygous (hTMEM106B(++)) animals, respectively. We showed that the increase in TMEM106B protein levels induced lysosomal dysfunction and age-related downregulation of genes associated with neuronal plasticity, learning, and memory. Increased TMEM106B levels led to altered synaptic signaling in 12-month-old animals which further exhibited an anxiety-like phenotype. Finally, we observed mild neuronal loss in the hippocampus of 21-month-old animals.

**Conclusion:**

Characterization of the first transgenic mouse model that overexpresses TMEM106B suggests that higher levels of TMEM106B negatively impacts brain health by modifying brain aging and impairing the resilience of the brain to the pathomechanisms of neurodegenerative disorders. This novel model will be a valuable tool to study the involvement and contribution of increased TMEM106B levels to aging and will be essential to study the many age-related diseases in which TMEM106B was genetically shown to be a disease- and risk-modifier.

**Graphical Abstract:**

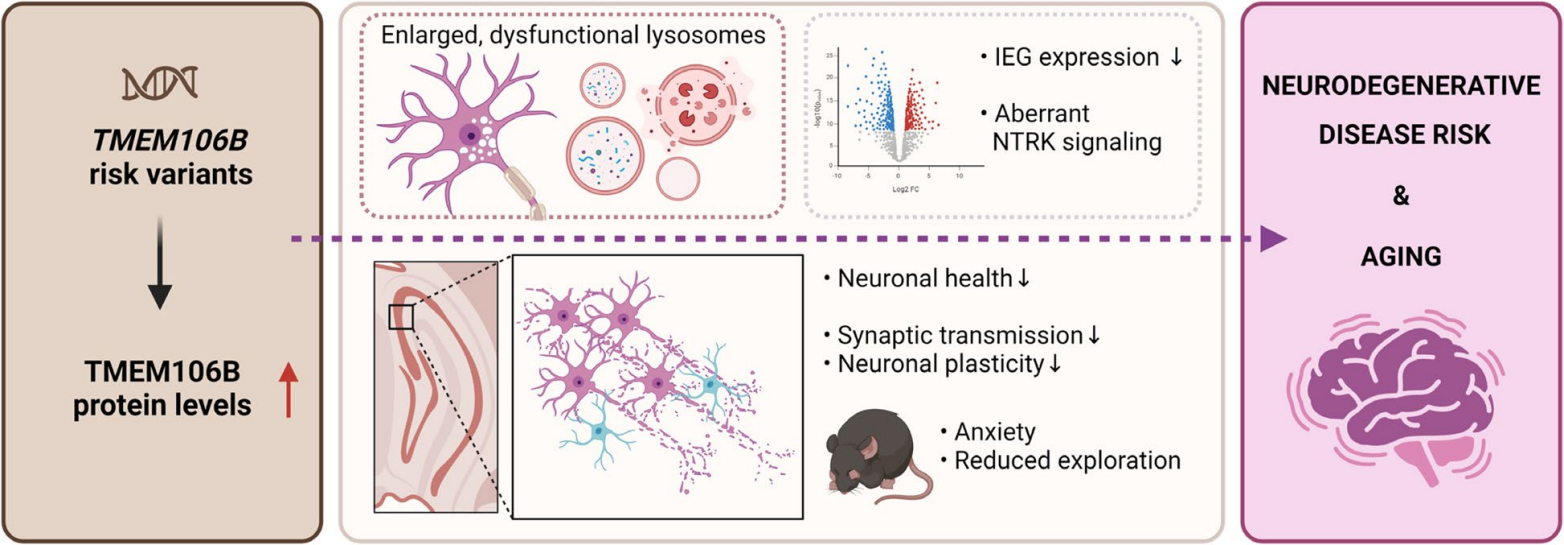

**Supplementary Information:**

The online version contains supplementary material available at 10.1186/s13024-025-00831-2.

## Background

Over the past decade, numerous studies have identified genetic variants in *TMEM106B* as important modifiers of disease risk in various neurodegenerative disorders, including Frontotemporal lobar degeneration (FTLD), Alzheimer’s disease (AD), Parkinson’s disease, hypomyelinating leukodystrophy, chronic traumatic encephalopathy (CTE), limbic-predominant age-related TDP- 43 encephalopathy (LATE), and hippocampal sclerosis in aging [1–11]. The genetic variation in *TMEM106B* has been associated with neuronal proportion, influencing neuronal vulnerability in general aging [12]. Indeed, TMEM106B has been linked to brain aging, even in the absence of known brain diseases[13]. TMEM106B’s importance in brain health is further highlighted by its connections to cognition and mood disorders, such as depression [3, 14–20]. In all these studies, a set of single nucleotide polymorphisms (SNPs) in and around the *TMEM106B* gene on chromosome 7p21 were found to be in high linkage disequilibrium, resulting in two common *TMEM106B* haplotypes in the human population. Since it is unclear which variant within the haplotype is functionally responsible for modulating disease risk, they are collectively referred to as either the risk or protective haplotype. The more frequent of these two haplotypes has consistently been associated with an increased risk of developing neurodegenerative diseases and poor brain health. A recent study shows that the protective *TMEM106B* haplotype is enriched in cognitively healthy centenarians as opposed to individuals with AD [21], further highlighting the importance of TMEM106B in healthy aging, cognition, and brain health.

Experimental evidence, mostly based on human studies, indicates that the variants on the *TMEM106B* haplotype influence disease risk by altering TMEM106B expression, with increased expression linked to the risk haplotype. Multiple studies found significantly elevated levels of *TMEM106B* mRNA and protein in *GRN* mutation carriers [22] and *TMEM106B* levels were shown to increase in the aging hippocampus, which was specific to carriers of the risk haplotype [23]. A non-coding variant (rs1990620) on the *TMEM106B* risk haplotype was suggested to modulate TMEM106B expression through changes in long-range chromatin-looping interactions [24]. In addition, there is one coding variant (rs3173615) encoding a Threonine to Serine change at amino acid position 185 (p.T185S) and in vitro it was shown that the protective isoform (S185) is associated with reduced TMEM106B levels, possibly due to less efficient N-glycosylation at residue 183 which may affect protein stability and degradation rate [25]. Moreover, *TMEM106B* contains miRNA- 132 and miRNA- 212 binding sites in its 3’ UTR, which inhibit TMEM106B expression upon binding [22]. In neurodegenerative conditions such as AD and FTLD-TDP, the expression of the microRNA- 132/212 cluster is decreased [26–29], suggesting an upregulation of TMEM106B expression in these diseases.

Considering the risk-modifying effect of *TMEM106B* is especially strong in FTLD-TDP caused by *GRN* mutations [9, 30], initial studies focused on reducing *Tmem106b* in *Grn* knock-out mouse models to evaluate whether reducing *Tmem106b* levels would ameliorate the disease phenotype. Multiple studies performed crossbreeding of *Tmem106b* knock-out mice with *Grn* knock-out mice, which surprisingly resulted in severe motor deficits and a worsening of the disease phenotype as compared to single *Grn* knock-out mice, leading to a premature death at 6–7 months of age [31–33]. Later studies also showed myelination deficits in the single *Tmem106b* knock-out model [34–36]. Since then, the field has consistently used the double knock-out model to study progranulin (PGRN) deficiency and the interplay between TMEM106B and PGRN. However, in human, the *TMEM106B* haplotypes have never been shown to associate with motor symptoms or motor neuron diseases. The only association to myelination and motor disease is with a rare dominant-negative mutation in *TMEM106B* (p.D252N), which leads to a loss of function of TMEM106B and a phenotype of hypomyelinating leukodystrophy [37, 38]. As such, knock-out of Tmem106b does not model the changes downstream of the genetic variation on the risk haplotype observed in neurodegenerative diseases and aging. Consequently, the precise function of elevated levels of TMEM106B, downstream of the genetic variants on the risk haplotype, is undetermined and it remains unclear how TMEM106B modulates disease risk and affects the aging process.

TMEM106B is a highly glycosylated, single-pass, type II transmembrane protein localized in the membrane of late endolysosomal compartments [39]. TMEM106B plays an important role in lysosome function which is demonstrated by the observation that both knockdown and overexpression of TMEM106B affect lysosomal morphology, pH, maturation, trafficking, and exocytosis [11, 37, 40–43]. While some studies described the cellular consequences of TMEM106B overexpression in in vitro cell models using transient overexpression, this is not representative of the physiological change in levels in the human brain and it is not informative of in vivo changes that may occur as a consequence of increased TMEM106B protein levels. There is only one study in which the researchers aimed to create a TMEM106B overexpression transgenic mouse model, however, this model failed to induce increased levels of TMEM106B due to the tight regulation of the protein [44]. Thus, the in vivo contribution of increased levels of TMEM106B to neurodegeneration and aging has not been assessed before.

In this study, we report on a novel transgenic mouse model that successfully overexpresses TMEM106B, with a 4- to 8-fold increase in TMEM106B protein levels in heterozygous (hTMEM106B(+)) and homozygous (hTMEM106B(++)) animals, respectively. We show lysosomal dysfunction in fibroblasts and primary cortical neurons derived from this model and identify age-related downregulation of genes associated with neuronal plasticity, learning, and memory. Consistent with these findings, we observe altered synaptic transmission in 12-month-old animals which display an anxiety-like phenotype. In addition, we observe mild neuronal loss in the hippocampus of 21-month-old animals. These findings support the hypothesis that the increase in TMEM106B, as a result of genetic variation in *TMEM106B*, negatively impacts brain health which modifies brain aging and impairs the resilience of the brain to neurodegeneration.

## Methods

### Mouse and cell lines

#### TMEM106B overexpression mice

Wild-type human TMEM106B (T185) cDNA was cloned into the CAG-Z-IRES-EGFP vector (provided by Yuji Mishina, University of Michigan) using AflII and SpeI sites. The CAG-Z-TMEM106B-IRES-EGFP construct was then was injected into fertilized oocytes from C57BL/6 female mice and implanted into pseudopregnant ICR mice to generate the Cre-inducible TMEM106B overexpression mice (Mayo Clinic Transgencic Core, Jacksonville). To generate TMEM106B overexpression mice, Cre-inducible TMEM106B mice were crossed with Meox2-Cre mice (Strain #:026858, The Jackson Laboratory). The mice were further back-crossed with C57BL/6 J wild-type mice to eliminate Meox2-Cre. Long-read Oxford Nanopore Sequencing determined that the ~ 4.5 kb construct integrated as a single copy at an intergenic region on mouse chromosome 17q23.2. Genotyping was performed on genomic DNA extracted from ear punches using PCR primers: 5’- GGAAAGTCCCTATTGGCGTT − 3’ (forward CAG promoter); 5’- ACCATCCCCAGAATCTGCTT − 3’ (forward wild-type); 5’-GGGGTAGGTGTACTGATGCA- 3’ (reverse wild-type). The ages of the mice used in each experiment are described and both male and female mice were used. Mice were housed in the animal facility at the University of Antwerp. All animal procedures performed were in accordance with EU (EU Directive 2010/63/EU for animal experiments) and local regulations and were approved by the Ethical committee for animal testing and the animal welfare body of the University of Antwerp.

#### Mouse embryonic fibroblasts

Mouse embryonic fibroblasts (MEFs) were prepared from embryos at E13.5 from heterozygous breeding pairs. The head, tails, and all red organs were removed, and the tissue was washed in PBS to remove excess blood. The tissue was then mechanically disrupted followed by two enzymatic digestion steps with Trypsin–EDTA 0.05% (Life Technologies, 25300–054) for 3 min at 37 °C. The cells were transferred to complete DMEM (10% FBS, 1% P/S, 1% L-Glutamine) and passed through a 70 µM cell strainer (VWR, 352,350) before plating. DNA was isolated from the tails using the Phire tissue kit (ThermoFisher) for genotyping purposes. The MEFs were kept for a maximum of 5 passages and experiments were performed within the same passage.

To quantify the vacuolization in MEFs, MEF cultures were imaged with an IncuCyte S3 (Essen Bioscience) live cell imaging system. Per genotype, 4 replicate wells (derived from one embryo) were used and phase contrast images from 8 positions per well were acquired. Image files (.tiff, 1408 × 1040) were exported from the IncuCyte software and spatially calibrated (0.62 µm/pixel) using the Fiji distribution of ImageJ [45]. To segment the vacuoles, a machine learning classifier was trained using Ilastik 1.4.0 software [46] that classifies every pixel to belong to either (i) background (no cell); (ii) vacuole or (iii) vacuole-negative part of cells (= cytoplasm or nucleus). Measurements of the number of pixels in each class were extracted from Ilastik binary segmentation masks using ImageJ [45].

#### Primary cortical neuron cultures

Primary cortical neurons were prepared from the cerebral cortices of E15-16 wild-type and heterozygous TMEM106B(+) embryos. We routinely combined the cortices of 2 or more embryos with the goal to obtain sufficient cells, and as such, all coverslips or wells were considered as a biological replicate. Briefly, the cortices were dissected in ice-cold HBSS buffer (Ca^2+^ and Mg^2+^ free; Thermofisher) supplemented with 1% penicillin/streptomycin, and incubated with protease solution (1:5 trypsin–EDTA 0.25% in HBSS) at 37 °C. The tissue was then mechanically disrupted, transferred to Neurobasal™ medium supplemented with 1X Glutamax, 2% B- 27 supplement, and 1% penicillin/streptomycin (all from Thermofisher), and passed through a 70 µM cell strainer. Cells were seeded in appropriate cell culture vessels precoated with 0.1 mg/ml poly-D-lysine (Thermofisher) at a density of 50,000 cells/cm^2^. Primary cortical cells were allowed to adhere for 1–2 h before the medium was replaced. The cells were maintained at 37 °C and 5% CO_2_ and half-medium changes were performed every 6–7 days.

### Cathepsin D activity assay

CTSD activity was measured using a CTSD activity assay kit (Abcam, ab65302) according to manufacturer’s instructions. Briefly, cells were lysed in CD Lysis buffer and centrifuged at 21,000 g for 5 min at 4 °C. Equal amounts of sample were loaded in triplicate and incubated overnight at 4 °C. The next day, CTSD substrate was added to the reaction and samples were incubated at 37 °C for 5 min. Fluorescence was measured with a Glomax Discover Microplate Reader (Promega, GM3500). Data is represented as relative fluorescence unit (RFU) normalized to the protein concentration (RFU/µg of protein). The protein concentration was measured using the BCA Assay (Pierce™ BCA Protein Assay Kit, Thermo Fisher Scientific, 23,227).

### Immunocytochemistry and live cell labeling of neuron cultures

Primary neurons were fixed with 4% PFA in PBS for 20 min at room temperature. Cells were kept at 4 °C until staining. Cells were incubated with blocking buffer (normal serum (1:500), 0.5% Triton-X- 100, 0.5% BSA in PBS) for 1 h at room temperature and incubated with primary antibody in PBT (0.02% Triton-X- 100, 0.5% BSA in PBS) overnight at 4 °C. The following primary antibodies were used: rabbit anti-TMEM106B (1:100, E7H7Z, Cell Signaling Technology), rat anti-LAMP1 (1:100, 1D4B, Santa Cruz Biotechnology), rabbit anti-EEA1 (1:100, 2411, Cell Signaling Technology), rabbit anti-LC3 (1:200, L7543, Sigma-Aldrich), chicken anti-MAP2 (1:2000, ab5392, Abcam). The next day, slides were incubated with Alexa Fluor conjugated secondary antibodies for 1 h at room temperature and Hoechst 33342 (1:20,000) was used to stain nuclei. Images were taken with a Zeiss LSM 900 confocal microscope. To label lysosomes by unquenching of DQ-BSA fluorescence, neurons were incubated for 3 h with DQ-Red BSA (Thermo Fisher, 10 μg/ml) before fixation. To assess autophagosome formation and potential autophagic identity of the vacuoles, neurons were treated with rapamycin (500 nM) and/or bafilomycin (50 nM) for 4 h before fixation.

#### LAMP1 and DQ-BSA intensity measurements

Fixed samples were imaged with an automated Nikon Ti2 microscope equipped with a Crest X-light V3 spinning disk using a CFI Plan Apochromat Lambda D 20X NA 0.8 objective lense and Photometrics Kinetics camera. Images of the neuronal cultures were automatically acquired at random positions in the sample using the Nikon NIS Elements software. To measure the intensity in a large number of neurons, the image datasets were analyzed using custom ImageJ [45] macro scripts to process the nd2 files in batch. In brief, all nuclei (neurons and glia) were segmented using Stardist [47] and stored in the ImageJ ROI manager. Nuclear segmentations were expanded by enlarging the nuclear ROIs by 10 pixels (3.3 µm) to obtain ROIs of the cells. Intensity measurements were performed after background subtraction (ImageJ rolling ball with radius 100 pixels). The data was filtered based on nuclear features to distinguish neurons from glial cells: only nuclei with an area between 60 µm2 and 140 µm2 and nuclear (Hoechst 33342) intensity values below a fixed threshold were included as neurons, thereby effectively excluding glial cells with smaller, more condensed nuclei. In total, 10,952 neurons originating from 12 wells/genotype and 8 images per well were measured for DQ-BSA; For LAMP1 and TMEM106B intensity measurements, 5 coverslips/genotype originating from 3 separate extractions were imaged, for each coverslip 10 image stacks were acquired at random positions.

#### Lysosomal mobility analysis

To label lysosomes for live cell imaging, primary cortical neurons were incubated 30 min with 100 nM Lysotracker Red (Thermo Fisher). After 30 min, the medium was changed to clear Neurobasal™ medium (12348017, Thermofisher) and neurons, maintained at 37 °C and 5% CO_2_ by an Okolab microscope incubator, were imaged with an automated Nikon Ti2 microscope equipped with a Crest X-light V3 spinning disk and using a CFI Plan Apochromat Lambda D 60X Oil NA 1.42 objective and Photometrics Kinetics camera. Images (2720 × 2720 pixels, 0.11 µm/pixel)) were captured at 1s intervals over 181 time points. Lysosomal mobility was measured using the TrackMate plugin in Fiji [45]. The Difference of Gaussians (DoG) detector was configured to detect all lysosome particles, and the SparseLAPTrackerFactory was used for linking detected spots into tracks. The tracking data, including individual spot coordinates and track details, were exported to CSV files for further analysis. Automation and batch processing via ImageJ macro scripts enabled high-throughput analysis of multiple images, ensuring robust and reproducible measurement of lysosomal mobility.

### Expansion microscopy

#### Sample preparation

Expansion microscopy was performed with a protocol largely based upon Chozinski et al. [48]. After standard immunofluorescence staining, the samples were crosslinked with 0.1 mg/ml ACX (11584017, Thermo Fisher Scientific) in PBS overnight at 4 °C. The samples were subjected to a gelation step using a mixture of 2 M NaCl (7647–14 - 5, Sigma-Aldrich), 2.5% (w/w) acrylamide (79–06 - 1, Sigma-Aldrich), 0.15% (w/w) N,N′-methylenebisacrylamide (110–26 - 9, Sigma-Aldrich), and 8.625% (w/w) sodium acrylate (7446–81 - 3, Sigma-Aldrich), 1 × PBS (11530486, Thermo Fisher Scientific). The polymerization was induced using 0.2% TEMED (110–18 - 9, Sigma-Aldrich) and 0.2% APS (17874, Thermo Fisher Scientific) and samples were left to polymerize for 1 h at 37 °C. After polymerization of the gels, the cover glasses were removed and the samples embedded in the gels were incubated for 30 min at 37 °C in a digestion buffer containing 0.5% Triton-X- 100 (T8787, Sigma-Aldrich), 1 × Tris–EDTA (93,283 - 100ML, Sigma-Aldrich), 0.8 M Guanidinium HydroChlodride (G3272 - 500G, Sigma life science), 8 U ml − 1 proteinase K (25530049, Thermo Fisher Scientific). The gels were then washed in high volumes (> 50 ml) of distilled water, which was exchanged at least three times to obtain maximal expansion of the gels. The expanded gels (4 × expansion factor) were trimmed and positioned in a 50-mm-diameter glass-bottom dish (GWST- 5040, WillCo Wells) and immobilized using 2% UltraPure LMP agarose (16,520–050, Invitrogen).

#### Quantification of lysosomal size

Expanded samples were imaged with a Zeiss LSM 900 confocal laser scanning microscope with Airyscan 2 detector using a C-Apochromat 63 × 1.2 NA water immersion objective. The Airyscan detector was used in SR- 4Y mode with default (‘auto’) Airyscan processing settings. Image z-stacks (encompassing the full depth of the sample) were acquired with optimal settings (2 × Nyquist sampling intervals) resulting in image voxels of 41 nm × 41 nm × 180 nm, corresponding to 10 nm × 10 nm × 45 nm when correcting for the four-time expansion of the sample.

The size of individual lysosomes was quantified using ImageJ [45] by manually delineating lysosome perimeters in their largest XY plane. Perimeter ROIs were saved and measured via the ImageJ ROI manager. To extract the diameter of a lysosome, we calculated the average between the min and max Feret diameter and corrected for (4x) expansion factor of the sample. We measured all distinguishable lysosomes in four cells of both genotypes, resulting in over 1600 lysosome diameter measurements in total.

### Transmission electron microscopy

For transmission electron microscopy, primary neurons grown on glass coverslips were immersed in a fixative solution consisting of 4% paraformaldehyde (28906, Thermo Fisher Scientific) and 2.5% glutaraldehyde (111–30 - 8, Sigma-Aldrich) in 0.1 M sodium cacodylate buffer pH 7.2 for 1 h at room temperature. After washing, cells were post-fixed in 1% osmium tetraoxide with potassium ferricyanide in 0.1 M sodium cacodylate buffer pH 7.2 at room temperature for 1 h. Afterward, samples were washed with ddH2O and subsequently dehydrated through a graded series of ethanol, including a bulk staining with 1% end concentration of uranyl acetate at the 50% ethanol step followed by embedding in Spurr’s resin. Ultrathin sections of a gold interference colour were cut using an ultramicrotome (Leica EM UC6) followed by a post-staining in a Leica EM AC20 for 40 min in uranyl acetate and for 10 min in lead stain at 20 °C. Sections were collected on formvar-coated copper slot grids and images were acquired with a JEM 1400plus transmission electron microscope (JEOL) operating at 80 kV.

### Western blot

Brain tissues were homogenized in ice-cold TBS using IKA® ULTRA-TURRAX tissue homogenizer (1:5 w/v) and adjusted to 1X RIPA buffer supplemented with protein and phosphatase inhibitors. Similarly, cell pellets were resuspended in 1X RIPA buffer. The suspensions were lysed on ice for 30 min with a brief vortex every 5 min. Cell lysates were sonicated for 5 min (Sonicator bath, VWR). Samples were centrifugated at 14,000 rpm for 15 min and supernatant was collected. The protein concentration was measured using the BCA Assay (Pierce™ BCA Protein Assay Kit, Thermo Fisher Scientific, 23,227). The samples were diluted in NuPAGE LDS sample buffer (Invitrogen, NP0007) supplemented with 5% β-mercaptoethanol (Sigma-Aldrich, M6250) and further denatured by boiling for 1–5 min at 95 °C. Electrophoresis was performed using NuPAGE Novex 4–12% Bis–Tris Gels (Invitrogen, NP0321) in NuPAGE MOPS SDS running buffer (Invitrogen, NP0001). Proteins were transferred onto an Immobilon PVDF membrane (Merck, IPFL00010). Primary antibodies included: mouse anti-hTMEM106B (1:1000, 60,333–1-Ig, Proteintech), rabbit anti-TMEM106B (1:1000, E7H7Z, Cell Signaling Technology), rabbit anti-TMEM106B fibrils (1:100, VIB_SB0051), rabbit anti-GFP (1:2000, 50,430–2-AP, Proteintech), rat anti-LAMP1 (1:1000, 1D4B, Santa Cruz Biotechnology), rabbit anti-CTSD (1:1000, AF1029, R&D Biosystems), rabbit anti-CTSB (1:1000, D1 C7Y, Cell Signaling Technology), mouse anti-NeuN (1:1000, MAB377, Merck), rabbit anti-GFAP (1:2000, ab7260, Abcam), rabbit anti-IBA1 (1:1000, 019–19741, Wako), mouse anti-Synapthophysin (1:5000, 17,750, Santa Cruz Biotechnology), mouse anti-PSD95 (1:2000, ab13552, Abcam), rabbit anti-TDP43 (1:1000, 10,782–2-AP, Proteintech), rabbit anti-GAPDH (1:20,000, GTX100118, Genetex). Band intensities were quantified using ImageJ (NIH).

### Quantitative PCR

RNA was extracted using Qiagen RNAeasy Plus mini kit, and equal amounts of RNA were reverse transcribed using the iScript™ complementary DNA (cDNA) synthesis kit. Real-time quantitative PCRs (qPCRs) were performed using SYBR green on a QuantStudio™ 6 Flex Real-time PCR system. The following primers were used: mouse Tmem106b (Fw: CGCGTGCGGTTTCTAGAGCAT, Rv: CCTCCCCGGGCTCTCAATGT), human TMEM106B (Fw: GGGCAAGAAAACCAACTG GTGGC, Rv: TCACGTCGATAGAGCGAGGGAA), Fos (Fw: GCATGGGCTCTCCTGTCAA, Rv: GGCACTAGAGACGGACAGATCTG), Arc (Fw: ATCTGTTGACCGAAGTGTCCAA, Rv: CCGACCTGTGCAACCCTTT), Egr2 (Fw: CGGGAGATGGCATGATCAAC, Rv: ACTCGGATACGGGAGATCCA), Gapdh (Fw: TGACCTCAACTACATGGTCTACA, Rv: CTTCCCATTCTCGGCCTTG), ActB (Fw: TGACGTTGACATCCGTAAAG, Rv: GAGGAGCAATGATCTTGATCT). Results were analyzed with the QuantStudio™ Real-time PCR Software using the comparative CT method. Data are expressed as 2^–ΔΔCT^ for the experimental gene of interest normalized to the housekeeping genes (Gapdh and β-Actin) and presented as relative quantity (RQ) relative to the control group.

### Brain histology and immunofluorescence stainings

Brains were dissected and fixed in 4% paraformaldehyde (PFA) in PBS for 24 h at 4 °C. Hemibrains were dehydrated in 70%, 95%, and 100% ethanols and chloroform, embedded in paraffin, and cut into 5 µm sections (Leica Histocut Rotary microtome). Paraffin sections were deparaffinized with xylene and rehydrated in a series of ethanol washes (100%, 95%, 70%). Antigen retrieval was performed by boiling in citrate buffer (pH 6.0) for 15 min and autofluorescence was quenched by incubating with Sudan Black (0.1% in 70% ethanol) for 10 min. Tissue slides were blocked in normal serum (1:250) in PBST for 1 h at room temperature and incubated with primary antibody overnight at 4 °C. The following primary antibodies were used: rabbit anti-TMEM106B (1:100, E7H7Z, Cell Signaling Technology), rat anti-LAMP1 (1:100, 1D4B, Santa Cruz Biotechnology), mouse anti-NeuN (1:400, MAB377, Merck), rabbit anti-GFAP (1:1000, ab7260, Abcam), rabbit anti-IBA1 (1:250, 019–19741, Wako), rabbit anti-TMEM106B fibrils (1:100, VIB_SB0051). The next day, slides were incubated with Alexa Fluor conjugated secondary antibodies for 1 h at room temperature and Hoechst 33,342 (1:20,000) was used to stain nuclei. Immunostaining for TDP- 43 pathology was performed using the Ventana automated immunostainer, using primary antibodies recognizing phosphorylation-independent TDP- 43 (monoclonal rat, clone 12B4, own production raised against murine Tardbp aa 200–222) and S409/410 phosphorylation-specific TDP- 43 (monoclonal rat, clone 1D3) [49]. Histological assessment at 21 months old was performed on sagittal brain sections from 6 wild-type, 6 hTMEM106B(+), and 5 hTMEM106B(++) animals.

#### Microscopy and pathology quantification

Slides were imaged using a Nikon Ti2-E microscope equipped with a Kinetix camera (Photometrix) and D‐LEDI Fluorescence Illumination using either a CFI Plan Apochromat Lambda D 10X NA 0.45 or a CFI Plan Apochromat Lambda D 20X NA 0.8 objective lense. Entire brain sections were imaged by acquiring overlapping tile images and stitching on selected regions using the Nikon NIS Elements software (‘large image acquisition’).

QuPath version 0.4.3 software [50] was used for visualization and quantification of the presence and intensity of different markers. We manually delineated brain regions based on the Allen Mouse Brain Atlas[51], including cortex, caudoputamen, thalamus, cerebellum, and hippocampal region. Several sets of features were extracted to quantify the presence and intensity of pathology markers. To determine the area proportion positive for a marker (such as NeuN), we trained sparse labeling machine learning classifiers for segmentation (Qupath *addPixelClassifierMeasurements* module). In addition, we segmented all cells (using the nuclear Hoechst 33342 signal and subsequent morphological expansion) and extracted the proportion of cells positive for the marker channel (based on intensity thresholds) (QuPath *PositiveCellDetection* plugin).

### RNA sequencing and bioinformatic data analysis

Brains from 8 mice were harvested from 15-month-old hTMEM106B mice (4 wild-type and 4 hTMEM106B(+)). RNA was extracted from cerebral hemibrains using the RNeasy® Plus Mini Kit (#74,136; Qiagen) kit. After measuring RNA integrity number (RIN) with a 2100 Bioanalyzer using the RNA Nano Chip (Agilent), samples were sequenced at Mayo Clinic’s Genome Analysis Core using Illumina HiSeq 4000 at 10 samples/lane. Reads were aligned to the reference genome (mm10) using HISAT2 [52]. Gene-level expression was quantified using HTSeq count, in reverse stranded mode, using the GTF file for mm10 from Ensembl [53]. Differential expression analysis (DEA) was performed using DESeq2[54]. DEA was corrected for sex and genes with fewer than 4 samples with at least 10 supporting reads were excluded. Benjamini–Hochberg procedure (BH step-up procedure) was performed to adjust for multiple testing and control for false discovery rate. Differentially expressed genes (DEGs) were defined by the threshold of BH step-up *P*-values < 0.05 and a log2 FC > ± 0.4. EnrichR was used for pathway analysis [55]; using “Reactome 2022” gene sets to determine pathway enrichment. Interaction network analysis between DEGs was performed using STRING protein–protein interaction network analysis. Visualizations were generated using ggplot2.

### Electrophysiology

Coronal hippocampal slices were made from 12-month-old TMEM106B(+) and WT animals. Brain slices (350 µm) were cut using a vibrating microtome (Microm HM650 V). Slices were prepared in aCSF cutting solution (65 mM sucrose, 85 mM NaCl, 2.5 mM KCl, 1.25 mM NaH_2_PO_4_, 7 mM MgCl_2_, 0.5 mM CaCl_2_, 25 mM NaHCO_3_, and 10 mM glucose, pH 7.2–7.4), bubbled with carbogen gas (95% O2/5% CO2) and immediately transferred to a storage chamber containing aCSF storage solution (130 mM NaCl, 3.5 mM KCl, 1.2 mM NaH_2_PO_4_, 2 mM MgCl_2_, 2 mM CaCl_2_, 24 mM NaHCO_3_, and 10 mM glucose, pH 7.2–7.4, at 32 °C), and bubbled with carbogen gas until used for recording.

Field recordings were performed in slices transferred to an interface chamber (Scientific Systems Design, Hofheim, Germany) continuously superfused with aCSF with the same composition as the storage solution and constantly bubbled with carbogen gas (32 °C and perfusion speed of 2 ml/min). Recordings were made using glass pipettes, pulled from standard wall borosilicate glass capillaries, and containing aCSF using a Multiclamp 700 A amplifier (Axon Instruments, Molecular Devices, CA, USA). Field excitatory postsynaptic potentials (fEPSPs) were evoked every 10 s using a Tungsten stimulation electrode (FHC inc., stimulation range: 100–500 µA) and a DS3 stimulator (Digitimer). Both stimulation electrode and recording glass pipettes were placed in the stratum radiatum of CA1. Long-term potentiation (LTP) was induced using two trains of stimulation at 100 Hz each lasting 1 s given with a 1 s interval. Paired pulse stimulation consisted of two consecutive stimulations given at 20 Hz. Recordings were filtered at 4 kHz and acquired at 10 kHz using a X-series USB- 6341 A/D board (National Instruments, Texas, USA) and WinWCP software (University of Strathclyde).

Data were analysed offline using custom written programmes in Igor Pro (Wavemetrics). fEPSPs were seen as downward deflections in recordings and their peak amplitude was measured. For plots, the fEPSP amplitudes post train stimulation were normalised relative to baseline and averaged per genotype.

### Behavioral testing

#### Morris water maze

The Morris water maze (MWM) experimental paradigm assesses hippocampus-dependent visual-spatial learning and memory. In brief, the setup consists of a circular pool (150 cm diameter) filled with opacified water kept at 25 °C. The maze is surrounded by invariant visual cues. During the training phase, a round platform is placed just below the water surface that mice will have to reach within 120 s. After reaching the platform or after being placed on the platform, mice have to remain there for 10 s before being returned to the home cage. A total of 8 trial blocks consisting of 4 trials with an inter-trial interval of 15 min is applied during training. Two trial blocks per day are applied (inter-block interval 4 h). During the probe trial (4 days after the final training day) the platform is removed and mice swim freely for 100 s. Trajectories are recorded using a video-tracking system (Ethovision XT, Noldus, The Netherlands). During the training phase, the escape latency to the platform and path length are measured. During the probe trial, performance is expressed as the percentage of time spent in each quadrant of the MWM, and the number of crossings over the previous platform’s position.

#### Open field test

The open field (OF) test assesses spontaneous locomotion and exploration of a novel environment. During the dark/active phase, mice are placed in a brightly lit arena (50 × 50 cm2). During 10 min, a video tracking system (Ethovision XT) records trajectories and parameters include total path length, time spent in the center circle, and time spent in the corners, indicative of anxiety-related thigmotaxis.

#### Elevated plus maze

The elevated plus maze (EPM) is a cross-shaped maze approximately one meter from the ground that consists of two open and two closed arms. During the dark/active phase, activity of the mice in the maze is recorded for 10 min (Ethovision XT). Parameters include e.g. total path length, time spent and number of entries in open vs. closed arms, latency to first open arm entry, and allow assessment of anxiety-related behavior.

#### Passive avoidance learning

Passive avoidance learning was assessed using a compartmentalized step-through box during the dark (active) phase of the light/dark cycle. The setup consisted of a brightly lit compartment connected to a dark compartment via a sliding door. The mice were placed in the brightly illuminated compartment, and the sliding door connecting the compartments was opened after a period of 5 s. Upon complete entry into the dark compartment (four-paw criterion), the mice received a mild foot shock (0.3 mA for 1 s). Exactly 24 h later, the latency to re-enter the dark compartment was timed up to 300 s, and the percentage of animals that did not reach this criterion was compared between experimental groups.

#### Contextual fear test

During the test, an aversive, unconditioned stimulus (an electric shock), is paired with a conditioned stimulus (the experimental chamber) to elicit a freezing response, a reliable measure of fear in rodents. On the first day the animals were placed in the testing chamber (22.5 cm wide × 32.5 cm long × 33.3 cm high Plexiglass cage with a grid floor) and were allowed to acclimate for 5 min. On day 2 they were first allowed to explore the testing chamber for 2 min (pre-US score). After this exploration, a 30 s tone was delivered with a buzzer (frequency: 2150 ± 200 Hz, Star Micronics, Piscataway, USA). This auditory cue or conditioned stimulus (CS), was followed by a 2 s, 0.35-mA foot shock, which served as the unconditioned stimulus (US). Again the mice were allowed to explore for 2 min. A second pairing of the CS and US was presented after these 2 min, followed by another 30 s exploration (post-US score). Twenty-four mins later the animals were returned to the testing chamber for 5 min exploration in the same context as the previous day (context score). Ninety minutes later the animals were returned to the test chamber, but now the grid floor was hidden with a Plexiglass plate and sawdust to alter the context of the testing chamber. The animals were observed for 6 min. During the first 3 min no stimulus was delivered (pre-CS score). During the next 3 min phase the auditory cue was delivered (CS score).

Under the different conditions animals were scored for freezing every 10 s, leading to a maximum score of 12 bouts of freezing during baseline trials, 21 during the shock trials, 30 during the context trials, and both 18 for the pre-CS and CS trials. A freezing score was calculated by expressing the number of observed freezing bouts as the percentage of freezing bouts versus the total number of bouts in each of the five trial blocks.

#### Novel object recognition

The NOR test was performed to assess recognition memory and was performed during four consecutive days with a regular day/night cycle with standardization of time on each day. On the first two days of the protocol, mice were individually habituated to an empty arena (40 cm × 24 cm) during 10 min. On the third day (familiarization phase), two identical objects were placed 10 cm apart in the center of the arena and mice were allowed to freely explore the cage and objects for 5 min. On the fourth day (novel object phase) one object was replaced with a novel object (different color and shape, but similar in size). Mice were then placed in the arena and again allowed to explore for 5 min. Trajectories and nose-point locations were recorded. Exploration time was defined as the time during which the nose-point was directed towards one of the objects with a proximity of 3 cm. The recognition index (time spent exploring novel object divided by total time exploring both objects) was calculated as a measure of recognition. Time spent investigating each object was scored using the behaviour tracking software (Ethovision).

#### Depression-related tests

Depression-related symptoms in rodents can be evaluated by means of stress models where the animal is faced with a stressful, inescapable situation that has been suggested to engender a state of behavioural despair, akin to the hopelessness manifest in clinical depression [56]. The Porsolt forced swim test (FST) measures the time spent swimming versus the time spent floating in a tall cylinder filled with water; a glass 5 L cylinder (27 × 16 cm2; height × diameter), filled to a depth of 15 cm with water maintained at 25 ± 2 °C is used. The observation period lasts 6 min, during which cumulative immobility is scored. In the tail suspension test (TST), the mice are suspended by the tip of their tail to a rod with adhesive tape at 60 cm above tabletop. Immobility was defined as “hanging passively and completely motionless” and the observer recorded the cumulative immobility time versus the time that they struggled during the 6 min observation period.

A core symptom of depression is anhedonia, defined as the decreased capacity to experience pleasure. This feature is modelled in rodents as a decrease in responsiveness to rewards, such as a sweet sucrose solution. For the sucrose preference test, mice were isolated in small cages (22.5 cm × 16.7 cm × 14 cm; length × width × height). They each had free access to two drinking bottles, one filled with 250 mL tap water and the other with a 2% sucrose solution. Prior to testing, there was a 48 h adaptation period to habituate to the different types of fluid. The mice were subsequently deprived from food and liquids for 3 h. During the next 24 h, there was free consumption of water and 2% sucrose solution in the presence of ad libitum food. Fluid intake was measured by weighing the drinking bottles. Sucrose preference was calculated as the ratio of the sucrose solution intake over the total fluid intake.

The behavioral tests were performed in the following order: MWM, NOR, CF, FTS, TSP, OF, EPM, PA, SPT for male mice; and NOR, MWM, FTS, TSP, CF, PA, OF, EPM, SPT for female mice.

### Statistical analysis

In all experiments, data is represented as mean ± SEM. One-way or two-way ANOVA (or non-parametric variant) followed by Tukey’s multiple comparison test was used to test for statistical significance between multiple groups. Student’s t-test or Mann–Whitney U test was used to compare between two groups. All statistical analyses were performed using GraphPad Prism software (GraphPad Software, San Diego, CA). *P*-values < 0.05 were considered statistically significant.

## Results

### Design, development, and validation of TMEM106B overexpression model

**Fig. 1:**
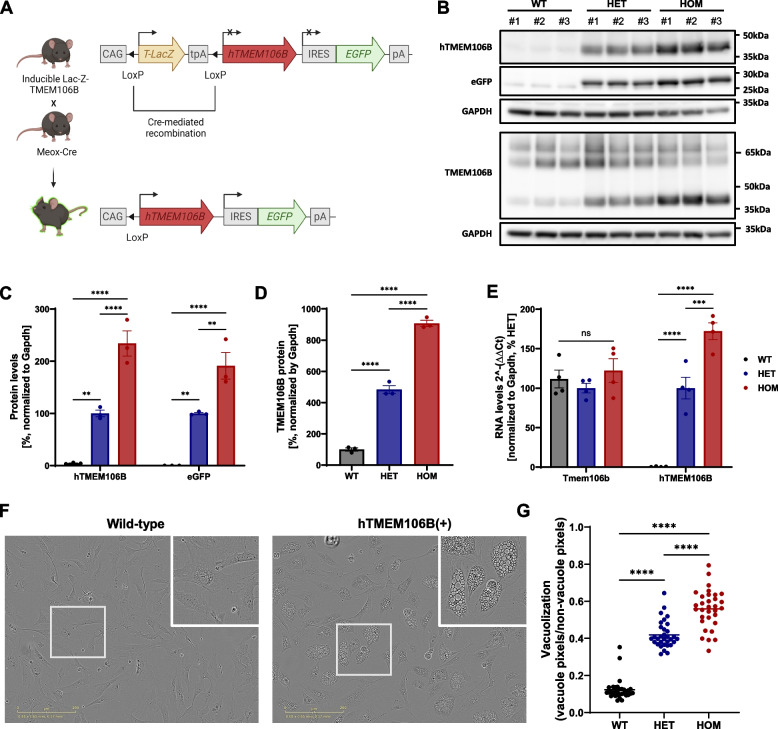
Development and validation of TMEM106B overexpression model. **A** Design of the transgene and induction of transgenic expression using Cre-mediated recombination. **B** Western blot of hemibrain lysates of 6-month-old animals using a human-specific anti-TMEM106B antibody (60,333–1-Ig, Proteintech), eGFP antibody (50430–2-AP, Proteintech), and anti-TMEM106B (E7H7Z, Cell Signaling Technology) (*n* = 3/genotype). **C** Quantification of the Western blot shows expression of the transgene, with a 2-fold increase in homozygous animals relative to heterozygous animals. **D** Quantification of total TMEM106B protein levels shows an approximately 4-fold to 8-fold overexpression for heterozygous and homozygous animals, respectively. **E** qPCR analysis of hemibrain lysates of 6-month-old animals using mouse-specific and human-specific qPCR primers shows expression of the transgene without downregulation of mouse *Tmem106b* (*n* = 4/genotype). **F** phase-contrast images of MEFs showing large vacuolar structures and **G** quantification of the vacuolar phenotype using pixel-based analysis. Per genotype, 4 replicate wells (derived from one embryo) were used and phase contrast images from 8 positions per well were acquired. Datapoints represent the average vacuolization ratio per image. Data represented as mean ± SEM. One-way ANOVA **, *P* < 0,01; ***, *P* < 0,001; ****, *P* < 0,0001

To study the functional consequences of elevated levels of TMEM106B on brain health in vivo, we generated Cre-inducible transgenic mice expressing human TMEM106B (hTMEM106B, T185 allele) under the control of the CAG promoter. Since previous studies have shown that fusion of an epitope tag on TMEM106B may disrupt its localization and function [57], we expressed the human TMEM106B transgene without an epitope tag. To generate mice expressing hTMEM106B in all epiblast-derived tissues, mice were crossed with mice expressing Cre recombinase under the Meox2 promoter[58]. Subsequently, mice were back-crossed with C57BL/6 J wild-type mice to eliminate any potentially harmful phenotypes associated with Meox2-loss (Fig. 1A).

Immunoblotting of hemibrain lysates from 6-month-old animals showed expression of hTMEM106B and eGFP, with a dose-dependent 2-fold increase in human TMEM106B protein levels in homozygous as compared to heterozygous animals. Considering a previously published TMEM106B overexpression model [44] failed to increase overall TMEM106B protein levels due to downregulation of mouse TMEM106B, we used a second antibody which is capable of recognizing both mouse and human TMEM106B and showed an approximate 4-fold and 8-fold overexpression in total TMEM106B levels in heterozygous (hTMEM106B(+)) and homozygous (hTMEM106B(++)) animals, respectively (Fig. 1B-D). qPCR analysis using human-specific and mouse-specific primers confirmed that mouse *Tmem106b* was not downregulated and confirmed the expression of the *hTMEM106B* transgene with a similar 1.5–2fold increase in homozygous versus heterozygous animals (Fig. 1E).

To validate whether a modest, stable, increase in TMEM106B was sufficient to induce lysosomal dysfunction, we extracted primary mouse embryonic fibroblasts (MEFs). Similar to what had been reported using transient overexpression [43], we found that the lumen of the MEFs contained many very large vacuoles (*P* < 0.0001). Quantification of the proportion of vacuolar regions showed that this phenotype was dose-dependent, with more vacuoles present in hTMEM106B(++) MEFs as compared to hTMEM106B(+) MEFs (*P* = 0.003) (Fig. 1F, G). Immunoblotting further showed an increase in LAMP1 and PGRN levels (Supplementary Fig. 1 A-B). Together this data confirmed the successful overexpression of TMEM106B and indicated that increased levels of TMEM106B induces lysosomal dysfunction.

### TMEM106B overexpression induces lysosomal dysfunction in neurons

**Fig. 2:**
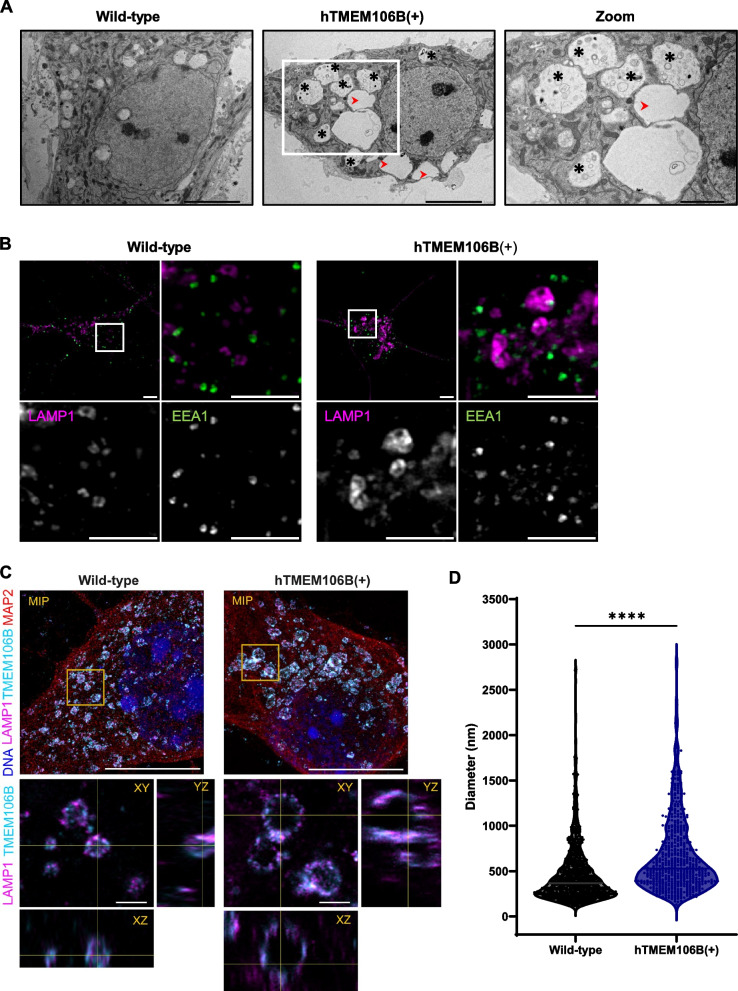
TMEM106B overexpression leads to large TMEM106B + vacuoles in primary neurons. **A** Numerous vacuoles were observed within the cytoplasm of hTMEM106B(+) neurons, including vacuoles containing fine material and membrane structures (asterisks) as well as empty, electron-clear vacuoles (red arrow heads). Scale bars (5 µm and 2 µm). **B** EEA1 staining shows normal EEA1 distribution and size and shows no colocalization with enlarged vesicles. **C** Expansion microscopy of wild-type and hTMEM106B(+) neurons. Scale bar (10 µm and 1000 nm). **D** The diameter of lysosomes was quantified for 4 cells/genotype. The large vacuolar lysosomes in hTMEM106B(+) neurons have a significantly higher diameter (*P* < 0.0001) and have fewer small (< 500 nm) lysosomes compared to wild-type neurons (*P* < 0.0001). Mann–Whitney U test

To assess the effect of increased levels of TMEM106B in a disease-relevant cell type, we next extracted mouse primary cortical neurons and evaluated lysosomal health. We used wild-type and hTMEM106B(+), but not hTMEM106B(++), for these experiments considering a ~ 4-fold increase in TMEM106B is physiologically most relevant when considering the expected increase in disease and during aging. Similar to what we observed in MEFs, hTMEM106B(+) primary neurons showed cytoplasmic vacuolation. Ultrastructural examination with TEM confirmed the presence of numerous electron-lucent cytoplasmic vacuoles with variable content, sizes, and shapes in hTMEM106B(+) neurons (Fig. 2A). While in few wild-type neurons similar structures could be observed, these were far less abundant and generally much smaller in size (Supplementary Fig. 2). A prior study described ultrastructurally similar vacuoles associated with TMEM106B expression [59]. We analyzed over 300 vacuoles in hTMEM106B(+) neurons. All vacuoles were enclosed by a single limiting membrane and exhibited diameters frequently exceeding 2 µm, with some reaching over 10 µm. Many vacuoles appeared fully electron-lucent and empty, while others contained sparse, heterogeneous content, including membrane-bound stretches of cytosol, multi-membrane structures, and partially degraded material (Supplementary Fig. 3). Collectively, the ultrastructural features of these vacuoles align with those of enlarged late autophagic vacuoles or aberrant endolysosomes.

Immunostaining for lysosomal marker LAMP1 confirmed the buildup of large lysosomal structures in the soma of hTMEM106B(+) neurons (Fig. 2B). In contrast, early endosomes (labeled with EEA1) were not visibly enlarged and did not accumulate (Fig. 2B), which further supports that disruptions caused by TMEM106B overexpression are restricted to the late endolysosomal pathway. To further characterize the vacuoles, we performed immunostaining with the autophagosome marker LC3 on wild-type and hTMEM106B(+) neurons which were either untreated or treated with rapamycin and/or bafilomycin. Similarly to what we observe with EM, the autophagosome marker LC3 staining further confirms that the majority of the vacuoles do not colocalize with LC3 and that only few vacuoles were associated with autophagosomes (Supplementary Fig. 4). To further explore the effect of TMEM106B on the lysosomal system, we stained for TMEM106B and the lysosomal marker LAMP1 and used expansion microscopy in combination with optical super-resolution (Airyscan) imaging to resolve the lysosomal structures far beyond the details obtainable with conventional light microscopy (Fig. 2C). Both endogenous and overexpressed TMEM106B is exclusively located at LAMP1-positive lysosomal membranes. Since we were able to resolve also a clear lumen in the smallest lysosomes (< 200 nm diameter), we compared the lysosomal size distributions between hTMEM106B(+) and wild-type neurons by measuring the diameter of all lysosomes in the cell. As expected, hTMEM106B(+) neurons on average have larger lysosomes (*P* < 0.0001). Interestingly, the proportion of the smaller lysosomes (< 500 nm diameter) is heavily reduced in hTMEM106B(+) compared to wild-type neurons (*P* < 0.0001) (Fig. 2A, supplementary movie 1–2), which indicates that in addition to inducing large lysosomal and/or vacuolar accumulations, TMEM106B expression also directly or indirectly affects the population of the smallest lysosomes.

**Fig. 3:**
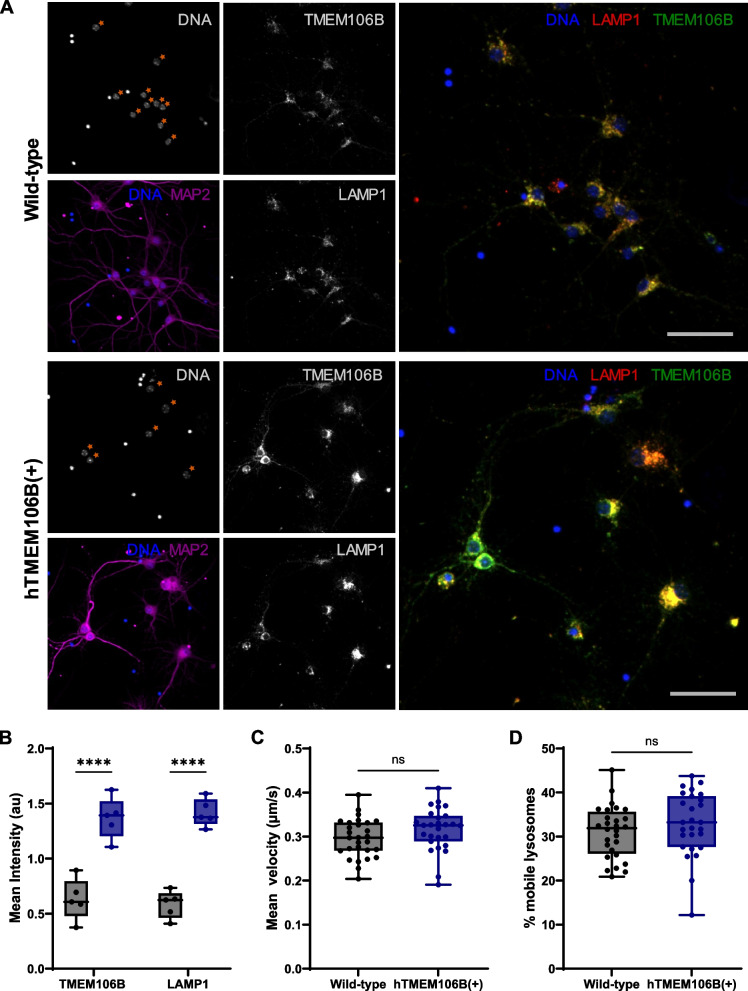
TMEM106B overexpression induces lysosomal dysfunction. **A** Representative images of hTMEM106B(+) and wild-type neurons stained for LAMP1 and TMEM106B, acquired with the same acquisition parameters, and displayed with identical brightness and contrast settings. Asterisks indicate example neurons that were included in the intensity measurement by discrimination from glial and/or dead cells based on nuclear features (see materials and methods for details). Scale bars (50 µm) and **B** Intensity measurements in wild-type and hTMEM106B(+) neurons show higher TMEM106B and LAMP1 intensity in TMEM106B overexpression neurons. Datapoints represent the average intensity per replicate (coverslip). Axonal lysosomal transport was evaluated by live cell imaging using Lysotracker. There was no significant difference in lysosomal mobility or speed between wild-type and hTMEM106B(+) neurons, quantified in **C-D**. Datapoints represent the average mobility or speed of tracks in one image field. Data represented as mean ± SEM. T-test *, *P* < 0,05; **, *P* < 0,01; ***, *P* < 0,001; ****, *P* < 0,0001

**Fig. 4:**
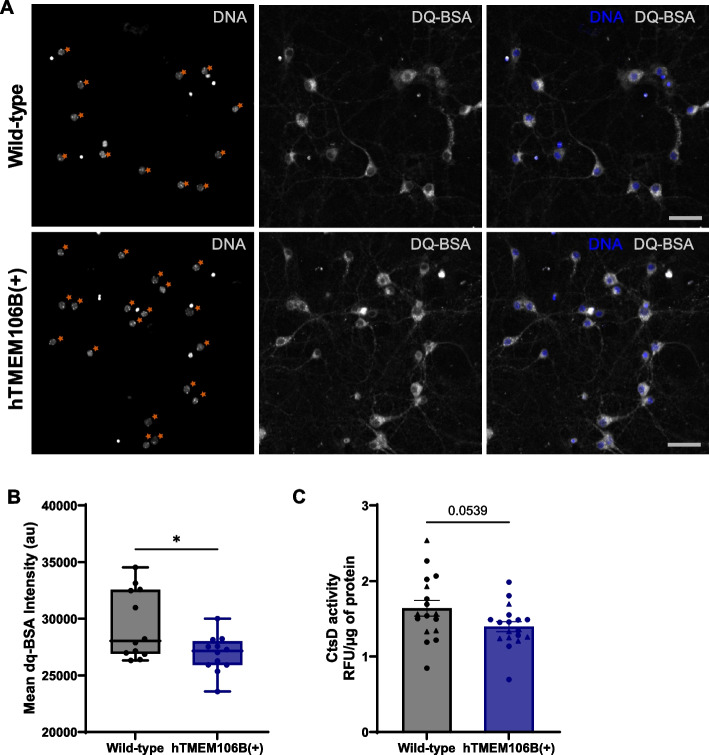
TMEM106B overexpression impairs lysosomal function. **A** Representative images of hTMEM106B(+) and wild-type neurons stained with DQ-BSA, acquired with the same acquisition parameters, and displayed with identical brightness and contrast settings. Asterisks indicate example neurons that were included in the intensity measurement by discrimination from glial and/or dead cells based on nuclear features (see materials and methods for details). Scale bars (50 µm) and **B** Intensity measurements in wild-type and hTMEM106B(+) neurons show lower DQ-BSA intensity in TMEM106B overexpression neurons (*P* = 0.018). Datapoints represent the average DQ-BSA intensity per replicate (well). **C** Proteolytic activity was further evaluated using a cathepsin D activity assay, showing a suggestive decrease in cathepsin D activity (*P* = 0.054). Data represented as mean ± SEM. T-test *, *P* < 0,05; **, *P* < 0,01; ***, *P* < 0,001; ****, *P* < 0,0001

Next, we assessed lysosomal health by evaluating lysosomal mobility, degradation capacity, and enzymatic activity. hTMEM106B(+) neurons showed higher TMEM106B and LAMP1 intensity (Fig. 3A, B). We did not observe a significant difference in lysosomal mobility or speed between wild-type and hTMEM106B(+) neurons (Fig. 3C, D). However, there was a significant decrease in DQ-BSA intensity (*P* = 0.018) and a suggestive decrease in cathepsin D activity (*P* = 0.054) (Fig. 4). Together with the observation of large structures containing undegraded material using EM these results suggest that hTMEM106B(+) neurons accumulate aberrant large lysosomal accumulations and have fewer functional and proteolytically active lysosomes.

### TMEM106B overexpression leads to age-related downregulation of immediate early genes

**Fig. 5:**
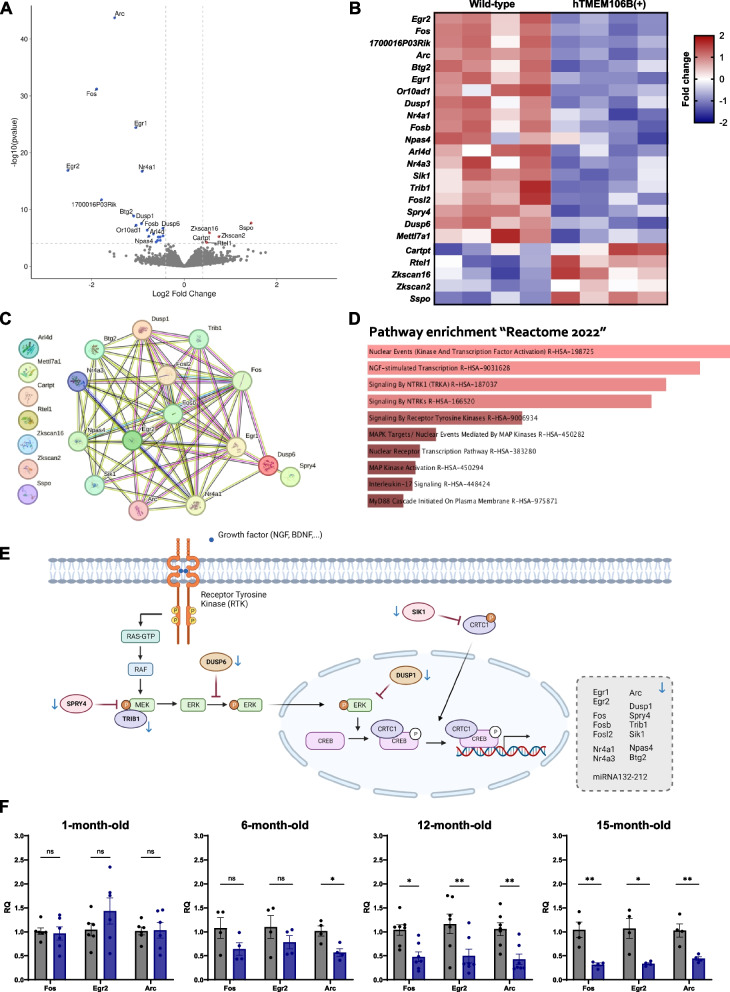
TMEM106B overexpression leads to age-related downregulation of immediate early genes. We performed bulk RNAseq on the cerebral hemibrain of 15-month-old animals (hTMEM106B(+) and wild-type (*n* = 4/genotype). **A** Volcano plot and **B** heatmap of differentially expressed genes using DESeq2. **C** STRING-DB Interaction network of DEGs shows clear interaction between most DEGs, which are well-known immediate early genes (IEGs). **D** Pathway enrichment analysis using Enrichr shows enrichment of the DEGs in neurotrophic tyrosine receptor kinase (NTRK) signaling. **E** Visual representation of the NTRK signaling pathway, highlighting the involvement and function of the identified DEGs in the pathway. Figure created with Biorender. **F** qPCR validation of top three differentially expressed genes across different age groups (*n* = 4–7/genotype) shows that there is no difference in expression in 1-month-old animals. The downregulation of IEGs is becoming apparent at 6 months of age with a significant downregulation of Arc in hTMEM106B(+) mice, which further progresses in a significant decrease in 12-month-old animals and 15-month-old animals. This data shows that the downregulation of IEGs is age-related and not present from birth. Data represented as mean ± SEM. Two-way ANOVA *, *P* < 0,05; **, *P* < 0,01

To gain insight into the effect of increased levels of TMEM106B in vivo, we performed bulk RNA sequencing on the cerebral hemibrain of 15-month-old hTMEM106B(+) and wild-type animals (Fig. 5A, B). Surprisingly, there was no upregulation or downregulation of lysosomal genes. There were only five upregulated genes (S*spo*, *Cartpt*, *Rtel1*, *Zkscan2*, and *Zkscan16*) and 19 downregulated genes (*Fos*, *Fosb*, *Fosl2*, *Arc*, *Egr1*, *Egr2*, *Nr4a1*, *Nr4a3*, *Btg2*, *Npas4*, *1700016P03Rik*, *Spry4*, *Trib1*, *Dusp1*, *Dusp6*, *Sik1*, *Or10ad1*, *Arl4 d, Mettl7a1*). Interaction network analysis revealed a strong link between 15 downregulated DEGs (Fig. 5C). Indeed, most of the downregulated genes are well-defined immediate early genes (IEGs): *Fos, Fosb, Fosl2, Arc, Egr1, Egr2, Nr4a1, Nr4a3*, and *Btg2*, as well as activity-regulated transcription factor *Npas4*. The neuroprotective genes *Btg2, Npas4*, and *Nr4a1* are also known as activity-regulated Inhibitor of Death (AID) genes [60, 61]. Interestingly, there was also downregulation of non-coding RNA 1700016P03Rik, encoding miR132 and miR212 [62, 63], which downregulates TMEM106B [22] and which has consistently been downregulated in neurodegenerative diseases [29].

Pathway enrichment analysis indicates enrichment of the DEGs in neurotrophic tyrosine receptor kinase (NTRK) signaling (Fig. 5D). Neurotrophins bind to highly homologous receptor tyrosine kinases which are encoded by NTRK genes, also known as tropomyosin receptor kinases (Trk). Upon binding, the Trk receptor dimerizes, autophosphorylates and then triggers different signaling pathways, including the mitogen-activated protein kinases (MAPKs) signaling pathway which results in the phosphorylation and activity of ERK1/2 and downstream phosphorylation and translocation of cAMP response element binding protein (CREB). Indeed, CREB is a well-known transcription factor that regulates IEG transcription and several of the remaining downregulated DEGs are known regulators of MAPK signaling. Specifically, *Spry4*, *Trib1*, and *Dusp6* are important for regulating MEK and ERK1/2 phosphorylation, *Dusp1* regulates the nuclear localization of pERK1/2, and *Sik1* regulates CREB [64–70]. These regulators, as well as miR132/212, are also downstream targets of transcription factor CREB which serves as a negative feedback loop (Fig. 5E). The downregulation of downstream targets (IEGs, miRNA132/212) as well as regulators of MAPK signaling in the brain of 15-month-old animals therefore suggest that increased TMEM106B levels may lead to aberrant neurotrophin signaling in vivo*.*

We validated the downregulation of the top three genes (*Fos*, *Arc*, *Egr2*) and assessed whether they are decreased developmentally, from birth, or whether the downregulation occurs during aging. qPCR analysis on the RNA from the cerebral hemibrain of 1-month, 6-month, 12-month, and 15-month-old animals showed no difference in expression in 1-month old animals. However, downregulation of IEGs was present at 6 months of age with a significant downregulation of Arc in hTMEM106B(+) mice and non-significant decrease in *Fos* and *Egr2*, which further progressed to a significant decrease of *Fos*, *Arc*, and *Egr2* in 12-month-old and 15-month-old hTMEM106B(+) animals (Fig. 5F). This data suggests that the downregulation of IEGs is age-related and not present from birth.

### TMEM106B overexpression alters synaptic transmission in the hippocampus

**Fig. 6:**
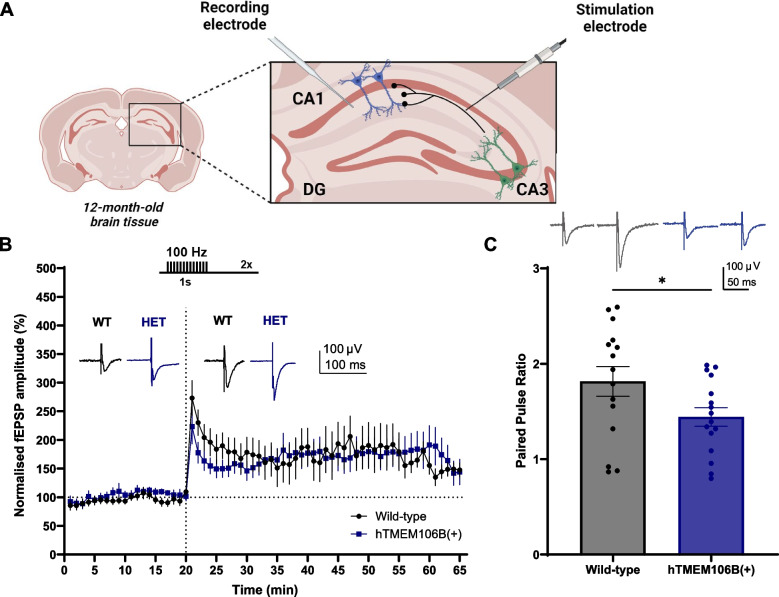
TMEM106B overexpression alters synaptic transmission in the hippocampus. **A** Graphical representation of the experimental set-up. We performed electrical stimulation of the Schaffer collateral pathway in the hippocampal CA1 region of 12-month-old animals and assessed the ability of synapses to undergo long-term potentiation (LTP) after high-frequency tetanisation (100 Hz). Figure created with Biorender. **B-C** There was no signifcant difference in LTP between wild-type and hTMEM106B(+) animals. However, there was a significant decrease in baseline (pre-tetanus) paired-pulse ratio (*P* = 0.04), with hTMEM106B(+) animals exhibiting reduced paired-pulse facilitation. LTP; *n* = 11–14 brain slices/genotype. PPR; *n* = 15–16 brain slices/genotype. Data represented as mean ± SEM. T-test *, *P* < 0,05

Considering the importance of neurotrophic signaling and IEGs, especially *Arc*, in synaptic transmission and plasticity [71–73], we performed in vitro electrophysiology experiments in acute brain slices from 12-month-old animals. We performed electrical stimulation of the Schaffer collateral pathway in the hippocampal CA1 region and assessed the ability of synapses to undergo long-term potentiation (LTP) after high-frequency tetanisation (100 Hz). In addition, we studied their synaptic release properties using a paired-pulse stimulation protocol (Fig. 6A). Both wild-type and hTMEM106B(+) animals exhibited robust LTP. However, using a paired-pulse protocol we observed that both genotypes exhibited paired-pulse facilitation but that this was significantly lower in hTMEM106B(+) animals (*P* = 0.04) (Fig. 6B, C). These findings indicate that hTMEM106B(+) animals already exhibit significant alterations in presynaptic function but that at this age this does not significantly impact synaptic plasticity in this region of the hippocampus.

### TMEM106B overexpression causes anxiety-like phenotype in 12-month-old mice

IEGs are well known for their function in learning and memory and have been linked to psychiatric disorders and mood disorders such as depression [74, 75]. Additionally, *Cartpt* (encoding Cart, cocaine-and-amphetamine-regulated transcript), which was one of the upregulated DEGs in hTMEM106B(+) mice, is a key neuropeptide involved in behavior such as reward, reinforcement, anxiety, stress, and depression. Increased *Cartpt* expression was found to correlate with susceptibility to anxiety-like behavior after exposure to stress [76].

**Fig. 7:**
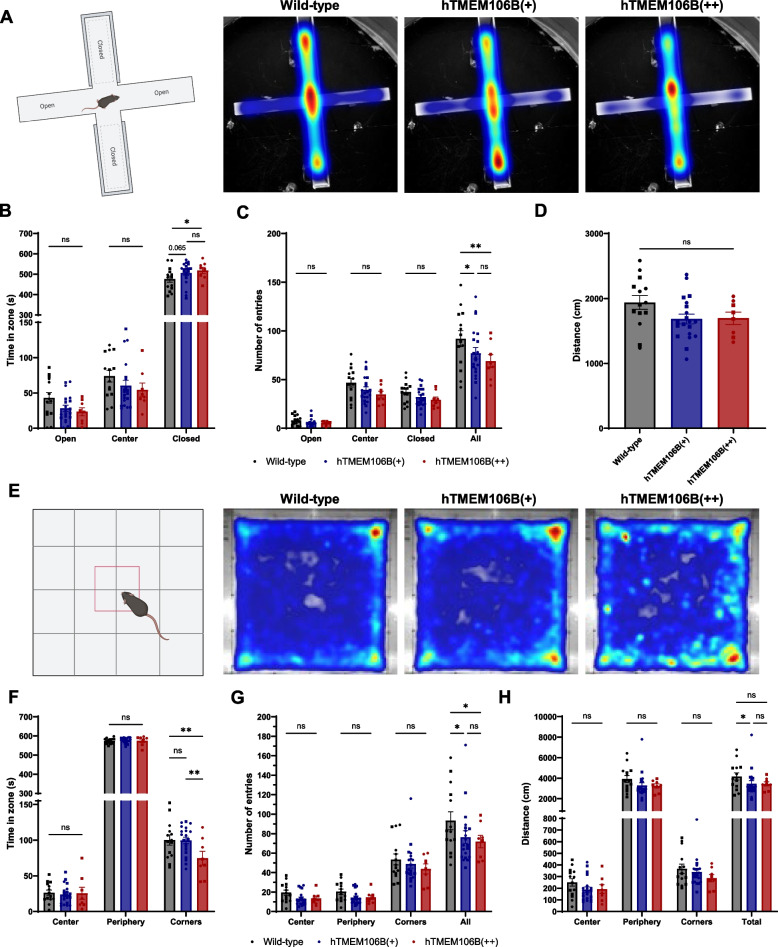
Increased TMEM106B levels leads to an anxiety-like phenotype in 12-month-old mice.** A** Graphical representation of the experimental set-up and composite heatmaps of wild-type (*n* = 14), hTMEM106B(+) (*n* = 20), and hTMEM106B(++) animals (*n* = 8) in the elevated plus maze. Figure created with Biorender. **B** Quantification of time spent in each zone, **C** the number of entries in each zone, and **D** the distance traveled within the maze. hTMEM106B(++) animals spent more time in the closed arms of the maze (*P* = 0.035) and hTMEM106B(+) animals showed a suggestive decrease in time spent in the closed arms (*P* = 0.065). hTMEM106B overexpression mice had fewer entries in each of the zones WT vs hTMEM106B(+), *P* = 0.023; WT vs hTMEM106B(++), *P* = 0.0044. **E** Graphical representation of the experimental set-up and composite heatmaps of wild-type, hTMEM106B(+), and hTMEM106B(++) animals in the open field test. Figure created with Biorender. **F** Quantification of time spent in each zone, **G** the number of entries in each zone, and **H** the distance traveled within the maze. hTMEM106B(++) mice spend less time in the corner of the box as opposed to wild-type (*P* = 0.008) and hTMEM106B(+) (*P* = 0.006) mice, the composite heatmap shows predominant localization of the hTMEM106B(++) mice to the sides and close to the corner of the box explaining the difference. TMEM106B overexpression animals show an overall decreased number of entries in the different zones of the maze (WT vs hTMEM106B(+), *P* = 0.025; WT vs hTMEM106B(++), *P* = 0.026) and travel less distance in each of the zones of the open field box. Together this data suggests that the overexpression animals are, overall, less explorative in the box and maze and show anxiety-like phenotype. Data represented as mean ± SEM. Two-way ANOVA, *, *P* < 0,05; **, *P* < 0,01

Therefore, as a next step, we performed an extensive set of behavioral tests on 6-month-old animals including the MWM, EPM, OF test, NOR, PA test, CF test, FST, and TST. However, we did not observe any differences between genotypes (data not shown). As a follow-up, we repeated the FST, TST, EPM, and OF test at 12 months of age in the same mice cohort. While there were no differences in the FST and TST (data not shown), we did observe a minor anxiety phenotype in the EPM, where hTMEM106B(++) animals spend more time in the closed arms of the maze (*P* = 0.035) and a non-significant trend for the hTMEM106B(+) animals (*P* = 0.065) as opposed to wild-type animals (Fig. 7A, B). Although we could observe that hTMEM106B(+) and hTMEM106B(++) mice appeared to, consequently, spend less time in the open arms and center of the maze, this was not statistically significant. Next, we also evaluated the number of entries in each zone of the maze and found that, overall, the overexpression mice have fewer entries in each of the zones, with a significant difference in the total number of entries in the overexpression mice as opposed to wild-type animals (WT vs hTMEM106B(+), *P* = 0.023; WT vs hTMEM106B(++), *P* = 0.0044) (Fig. 7C). This suggests that TMEM106B overexpression mice are less explorative.

In the OF test, we observed that hTMEM106B(++) mice spend less time in the corner of the box as opposed to wild-type (*P* = 0.008) and hTMEM106B(+) (*P* = 0.006) mice, which is inconsistent with what would be expected. However, the composite heatmap suggests a predominant localization of the hTMEM106B(++) mice to the sides and close to the corner of the box which explains this difference (Fig. 7E, F). Interestingly, the overexpression mice seem to travel less distance in each of the zones of the open field box, with a significant decrease in the total distance traveled across the different zones for hTMEM106B(+) animals as opposed to the wild-type animals (*P* = 0.044) (Fig. 7G, H). Similar to the EPM, there is an overall decrease in the number of entries (WT vs hTMEM106B(+), *P* = 0.025; WT vs hTMEM106B(++), *P* = 0.026). These differences are not explained by motor deficits as, even at the age of 24-months-old, the overexpression mice did not show any clear motor deficits and did not display hindlimb clasping. This data suggests that the overexpression animals are, overall, less explorative in the box and maze. Together with the increase in the amount of time spent in the closed arms of the EPM, this indicates that mice overexpressing TMEM106B have an anxiety-like phenotype at 12-months of age.

### TMEM106B overexpression leads to cell loss in the hippocampus of 21-month-old mice

**Fig. 8:**
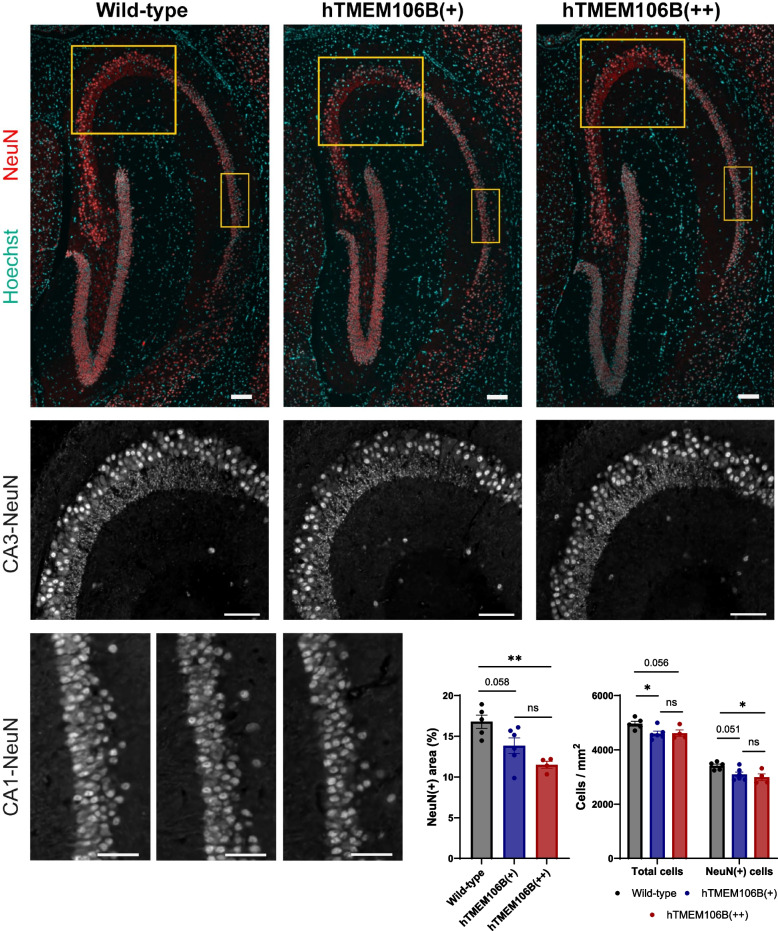
TMEM106B overexpression leads to cell loss in the hippocampus of 21-month-old mice. **A** Representative images of NeuN immunostaining of the hippocampal region of wild-type, hTMEM106B(+), and hTMEM106B(++) mice (*n* = 4–6/genotype) with zooms for the CA1 and CA3 regions. Scale bars (100 µm). **B** Quantitative measurements of neuronal loss included the quantification of the NeuN(+) area and quantification of NeuN(+) cells and total cells/mm^2^ (Hoechst). hTMEM106B(+) show a suggestive reduction in NeuN(+) cells (*P* = 0.051), a reduction in total cells/mm^2^ (*P* = 0.025), and a suggestive decrease in total NeuN(+) area (*P* = 0.058). hTMEM106B(++) show a reduction in NeuN(+) cells (*P* = 0.018), a suggestive reduction in total cells/mm.^2^ (*P* = 0.056), and a decrease in total NeuN(+) area (*P* = 0.0035). Data represented as mean ± SEM. ANOVA *, *P* < 0,05; **, *P* < 0,01

To assess whether TMEM106B overexpression causes lysosomal or neurological changes in the brain such as inflammation or neuronal cell death we evaluated both protein lysates as well as brain slices from 21-month-old animals. Consistent with the bulk RNA sequencing results, we did not observe changes in total lysosomal protein levels of LAMP1, CTSD, and CTSB. We also did not observe apparent changes in the levels of NeuN, GFAP, IBA1, or synaptophysin on immunoblot (Supplementary Fig. 5). While immunostaining of sagittal brain sections did not reveal gliosis or neuroinflammation in the brain using IBA1 or GFAP staining (Supplementary Fig. 6), we did observe mild neuronal loss in the hippocampus of hTMEM106B(+) and hTMEM106B(++) mice as shown by a reduction in NeuN(+) cells and total cells/mm^2^, and a decrease in the proportion of NeuN(+) area (Fig. 8). hTMEM106B(+) mice show a suggestive reduction in NeuN(+) cells (*P* = 0.051), a reduction in total cells/mm^2^ (*P* = 0.025), and a suggestive decrease in the proportion of NeuN(+) area (*P* = 0.058). hTMEM106B(++) mice show a reduction in NeuN(+) cells (*P* = 0.018), a suggestive reduction in total cells/mm^2^ (*P* = 0.056), and a decrease in the proportion of NeuN(+) area (*P* = 0.0035).

TDP- 43 aggregation and hyper-phosphorylation is a hallmark for FTLD and several other neurodegenerative disorders. However, we failed to detect TDP- 43 pathology in hTMEM106B overexpression mice (Supplementary Fig. 7). Recent studies identified that the C-terminal fragment (CTF) (AA120 - 254) of TMEM106B can form amyloid filaments in the brain of patients with a variety of neurodegenerative diseases as well as older neurologically normal individuals. We evaluated the presence of TMEM106B fibrils in 24-month-old mice using IHC and immunoblotting of the sarkosyl-insoluble fraction but failed to detect the presence of TMEM106B immunoreactive pathology both in wild-type and in hTMEM106B overexpression mice (Supplementary Fig. 8).

## Discussion

Genetic variation in *TMEM106B* is known to influence healthy aging and modify the risk and disease presentation of several neurodegenerative diseases. Current evidence shows that the risk haplotype is associated with higher levels of TMEM106B, both in aging and in disease. However, most studies have focused on *Tmem106b* knock-out models as this worsened the phenotype of *Grn* knock-out mice in initial studies. Importantly, however, results from these knock-out models merely underlined the tight regulation and importance of adequate levels of TMEM106B in the brain, and did not model the changes downstream of the risk haplotype observed in human neurodegenerative diseases and aging. This means that the contribution of elevated levels of TMEM106B, downstream of the genetic variants, to neurodegeneration and aging had not been studied.

Here, we generated Cre-inducible transgenic mice expressing hTMEM106B (T185 allele) under the control of the CAG promoter leading to a 4-fold (hTMEM106B(+)) or 8-fold (TMEM106B(++)) overexpression of TMEM106B as compared to the levels of endogenous mouse TMEM106B. This is in contrast to a prior model where, despite the expression of the human transgene, the total protein levels of TMEM106B were not changed. The prior model was only capable of inducing elevated levels of TMEM106B in aged *Grn* knock-out mice where it exacerbated the lysosomal pathology caused by PGRN loss [44]. Considering the demonstrated tight regulation of TMEM106B, we specifically chose to use a strong CAG promoter and subsequently activated expression in all epiblast-derived tissues by crossbreeding with mice expressing Cre recombinase under the Meox2 promoter.

Multiple studies have suggested a critical role for TMEM106B in regulating lysosomal function and alterations in TMEM106B levels have been shown to affect multiple aspects of lysosomal biology. Yet, these studies were limited by the use of transient overexpression which induces an extreme overexpression of TMEM106B and does not accurately reflect the increase in expression observed in aging and disease. We evaluated lysosomal health in primary cortical neurons from hTMEM106B(+) mice, which have a ~ 4-fold overexpression of TMEM106B, and show cytoplasmic vacuolation in the perinuclear region. Ultrastructural examination showed the presence of numerous vacuoles, including vacuoles containing undegraded fine material and membrane structures as well as fully empty, electron-lucent vacuoles. All vacuolar structures were enclosed by a single membrane and are positive for both LAMP1 and TMEM106B which supports that the enlarged structures are of late endolysosomal origin. Staining with the autophagosome marker LC3 staining further confirmed that the majority of the vacuoles do not colocalize with LC3 and that only few vacuoles were associated with autophagosomes. We further showed that hTMEM106B(+) neurons have fewer small, functional lysosomes and that the enlarged lysosomes are less proteolytically active. TMEM106B was shown to interact with MAP6 to control lysosomal trafficking, with knockdown of TMEM106B increasing retrograde lysosomal trafficking in dendrites [42]. However, we did not observe a significant difference in axonal lysosomal mobility between wild-type and hTMEM106B(+) neurons suggesting that lysosomal trafficking deficits may be specific to knockdown of TMEM106B.

To determine the in vivo effect of elevated TMEM106B levels, we performed bulk RNA sequencing on the cerebral hemibrain of 15-month-old animals. We show age-related downregulation of immediate early gene expression in hTMEM106B(+) mice. We also found downregulation of the precursor of miR132 and miR212 which regulates TMEM106B levels and which have been shown to be downregulated in AD and FTLD-TDP [22, 26–29]. This suggests either a feedback loop between miR132/212 expression and TMEM106B expression levels or the presence of underlying (disease) processes in the hTMEM106B(+) mice which are similar to those in AD and FTLD-TDP. Pathway enrichment analysis showed enrichment of the identified DEGs in NTRK signaling. We hypothesize that increased TMEM106B levels may lead to altered trafficking and/or delayed degradation of Trk receptors due to the observed lysosomal dysfunction. It has previously been shown that overexpression of TMEM106B delays the degradation of endocytic cargoes, such as EGFR [57]. Our study is the first to associate increased TMEM106B levels with aberrant neurotrophin signaling in vivo which could potentially explain its genetic link to mood disorders such as depression.

Interestingly, we show a link between increased TMEM106B levels in the brain and changes in synaptic properties in the hippocampus. We observed a decrease in the paired-pulse ratio in the CA1 region of the hippocampus of 12-month-old animals which may indicate altered synaptic transmission, in particular a presynaptic defect. Considering we also observed a decrease in the RNA levels of genes strongly associated with neuronal activity and neuronal plasticity, these findings further support the potential disruption of these important pathways. We propose two distinct mechanisms (i) a direct effect through modulation of the release capability or availability of presynaptic vesicles and/or (ii) disrupted lysosomal calcium storage and release; which could both lead to decreased paired-pulse facilitation. It is currently still unclear whether these defects are related to an (unknown) direct function of TMEM106B or are downstream of the lysosomal dysfunction induced by TMEM106B overexpression. Yet, the relevance of our findings is substantiated by the prior observation that the TMEM106B haplotypes correlated with gene networks involved in synaptic transmission in gene co-expression network analyses in aged and diseased human brains [77]. Similarly, a recent study used gene set enrichment analysis to show that TMEM106B levels are negatively associated with synaptic signaling protein networks in the aging human hippocampus [23]. In our current study, by using a novel TMEM106B overexpression mouse model, we suggest that synaptic signaling and transmission could be a key misregulated pathway downstream of increased levels of TMEM106B. Our findings indicate that future studies should focus on dissecting the role of increased levels of TMEM106B in synaptic signaling with detailed characterization of the precise mechanism, preferably in a human model such as iPSC models.

Neurotrophin signaling, synaptic signaling, and IEGs are important in neuronal plasticity, learning, and memory. We performed behavioral tests in TMEM106B overexpression mice in 6-month-old animals, the earliest time-point in which we observed a difference in IEG expression levels. However, we did not observe any differences between TMEM106B overexpression and wild-type mice. We later repeated some of the behavioral tests at 12 months of age and observed an anxiety-like phenotype and reduced exploration in the overexpression mice. However, we could not repeat behavioral tests related to learning or memory as this requires naïve mice, which is a limitation of the current study. Next, we assessed brain health in 21-month-old animals using immunoblotting and immunofluorescent stainings. Interestingly, consistent with transcriptomic analysis, we did not observe any apparent differences in inflammation markers. However, we found that aged TMEM106B overexpression mice show hippocampal neuronal loss, which shows that TMEM106B levels affect neuronal health and is consistent with human studies showing that the TMEM106B haplotypes are associated with neuronal proportion [12].

Recent studies showed that the C-terminal fragment of TMEM106B can form filaments in human brain [78–81]. Yet, we did not observe TMEM106B fibrils in aged wild-type mice nor TMEM106B overexpression mice using the same methodology as we used in human studies [82, 83]. A recent study also failed to detect TMEM106B aggregates in aged wild-type and Tau mice, confirming our findings [84]. While we can not exclude the possibility that TMEM106B fibrils may be present but could not be detected with our antibody, it is more likely that the mice do not produce TMEM106B fibrils. Replicating disease-associated inclusions and protein aggregates has been challenging in many models of neurodegenerative diseases. However, our model supports and phenotypically recapitulates what has been observed in risk haplotype carriers in human studies including the misregulation of synaptic transmission and impaired neuronal health. The lack of very severe brain abnormalities supports the current notion that TMEM106B is primarily a disease modifier which acts as a second hit. The model described in this study does not have additional disease-related mutations, and therefore reflects the effect of increased TMEM106B levels on general brain health and aging rather than modeling its disease-modifying capabilities. However, even in the absence of disease, as shown in our current model, increased levels of TMEM106B affect brain health and disrupts normal brain physiology. This is consistent with what has been observed in human aging studies, where *TMEM106B* haplotype affects neuronal proportion and differential aging parameters [12, 13]. Furthermore, we have shown a critical role of TMEM106B in regulating lysosomal function and it is well known that proper lysosomal functioning is especially important in neurodegenerative disorders, which are typically characterized by the abnormal accumulation of disease proteins. In sum, these findings suggest that an increase in TMEM106B, as a result of genetic variation in *TMEM106B*, impairs the resilience of the brain to the pathomechanisms of neurodegenerative disorders. Our novel model will be a valuable tool to study the involvement and contribution of increased TMEM106B levels to aging and disease in the many age-related diseases in which TMEM106B was genetically shown to be a disease- and risk-modifier.

## Conclusion

We created the first transgenic mouse model that successfully overexpresses TMEM106B. We show that the increase in TMEM106B protein levels induces lysosomal dysfunction and identified age-related downregulation of genes associated with neuronal plasticity, learning, and memory. We observed altered synaptic signaling in 12-month-old animals and further show that 12-month-old overexpression animals exhibit an anxiety-like phenotype. Finally, we show mild neuronal loss in the hippocampus of 21-month-old animals. This is the first study that models the contribution of TMEM106B, downstream of the disease-associated genetic variants, to neurodegeneration and aging. Our findings support the hypothesis that the increase in TMEM106B impairs brain health which modifies brain aging and impairs the resilience of the brain to the pathomechanisms of neurodegenerative disorders. Our novel model will be a valuable tool to study the involvement and contribution of increased TMEM106B levels to aging and will be essential to study the many age-related diseases in which TMEM106B was genetically shown to be a disease- and risk-modifier.

## Supplementary Information

Additional file 1: **Figure S1.** TMEM106B overexpression leads to increased LAMP1 and PGRN levels in MEFs. (A) Immunoblot and (B) quantification of LAMP1, PGRN, and TMEM106B protein levels indicate elevated levels of LAMP1 and PGRN in MEFs derived from TMEM106B overexpression mice which confirms that increased levels of TMEM106B induces lysosomal dysfunction. Data represented as mean ± SEM. One-way ANOVA (n=3/genotype). *, *P* < 0,05; **, *P* < 0,01. The 15kDa band represents an N-terminal fragment of TMEM106B of unknown significance which appears to be human-specific but is otherwise uncharacterized. **Figure S2.** TEM images of wild-type and TMEM106B(+) neurons showing large cytoplasmic vacuoles. Ultrastructural examination with TEM confirmed the presence of numerous cytoplasmic vacuoles with variable content, sizes and shapes in hTMEM106B(+) neurons. While in few wild-type neurons similar structures could be observed, these were far less abundant and generally much smaller in size. Scale bars (5 µm). **Figure S3**. Ultrastructural characterization of aberrant vacuoles in TMEM106B overexpressing neurons. The electron-lucent cytoplasmatic vacuoles are enclosed by single membranes. Many vacuoles are largely empty, or contain only small irregularly shaped cytosolic components A,B,C). Cytoplasmic invaginations can penetrate the vacuoles (C,D), in rare cases resembling a network of cytoplasm stretches (E). Some vacuoles contain mostly degraded material (F), while others mostly hold a build-up of membranes and vesicles (G, H). Scale bars (1µm). **Figure S4.** The vacuoles do not colocalize with the autophagosome marker LC3. Representative images of wild-type and hTMEM106B(+) stained for LAMP1 and LC3. Neurons were treated with rapamycin (500nM) and/or bafilomycin (50nM) for 4 h to induce autophagosome formation. The majority of the vacuoles do not colocalize with LC3. Only few vacuoles were associated with autophagosomes. Scale bars (20µm). **Figure S5**. TMEM106B overexpression does not lead to apparent lysosomal or neurological changes in 21-months-old mice. (A) Immunoblot and (B) quantification of hTMEM106B and total TMEM106B protein levels in hemibrain lysates of 21-month old animals. (C) Immunoblot and (D) quantification of lysosomal proteins indicate normal levels of lysosomal proteins in the brain. (E) Immunoblot and (F) quantification of neural markers GFAP, SYN, NeuN, and IBA1 indicate normal levels of neural cell types. Data represented as mean ± SEM. One-way ANOVA. *, *P* < 0,05; **, *P* < 0,01; ***, *P* < 0,001; ****, *P* < 0,0001. **Figure S6.** TMEM106B overexpression does not lead to gliosis in 21-month-old mice. Representative images of (A) GFAP and (B) IBA1 immunostaining of sagittal brain sections of wild-type, hTMEM106B(+), and hTMEM106B(++) mice. Scale bar (2 mm). Quantitative intensity measurements of (C) GFAP and (D) IBA1 (*n* = 4-6/genotype). Data represented as mean ± SEM. One-way ANOVA. **Figure S7.** TMEM106B overexpression mice do not have TDP- 43 aggregates. (A) Immunoblot and (B) quantification of TDP- 43 protein levels in RIPA- and UREA-soluble fractions of hemibrain lysates of 21-month-old animals does not show increased amounts of TDP- 43 in the insoluble fraction. Data represented as mean ± SEM. One-way ANOVA (*n* = 3/genotype). (C) Immunohistochemistry with phosphorylation-independent TDP- 43 (12B4) antibody reveals predominant nuclear staining in wild-type and hTMEM106B(+) mice as illustrated in different brain regions. (D) Immunohistochemistry with S409/410 phosphorylation-dependent TDP- 43 (1D3) antibody. No specific immunoreactivity was observed in wild-type and hTMEM106B(+) mice as illustrated in various brain regions. Insert shows positive control for 1D3 immunohistochemistry with nuclear cytoplasmic inclusions in human postmortem FTLD-TDP case. Scale bar 50 µm. **Figure S8**. Wild-type and TMEM106B overexpression mice do not generate TMEM106B fibrils. (A) We evaluated the presence of TMEM106B fibrils in 21-month-old mice using the same methodology as we used in human studies [82, 83]. (B) Immunoblot of the insoluble fraction does not show TMEM106B immunoreactive pathology in either wild-type or hTMEM106B overexpression mice. (C) Representative images of immunostainings of the cortex of 21-month-old Tmem106b knock-out, wild-type, and hTMEM106B(+) animals using VIB SB0051 fibril-specific antibody. All cases showed a similar pattern with staining of vascular endothelial cells and some small glia with morphology of microglia, that was interpreted as non-specific. None of the animals in any of the groups showed pathological accumulation of TMEM106B CTF, similar to what is seen in human cases.

Additional file 2. The additional movie files show a 3D rendering of primary neurons that were expanded and immunostained with anti-MAP2, anti-LAMP1and anti-TMEM106B antibodies. The videos show enlarged lysosomes and cytoplasmic accumulations in hTMEM106B(+) neurons (movie 2) as opposed to wild-type neurons (movie 1).

Additional file 3. The additional movie files show a 3D rendering of primary neurons that were expanded and immunostained with anti-MAP2, anti-LAMP1and anti-TMEM106B antibodies. The videos show enlarged lysosomes and cytoplasmic accumulations in hTMEM106B(+) neurons (movie 2) as opposed to wild-type neurons (movie1).

Additional file 4

## Data Availability

The data supporting the conclusions of this article is included within the article and its additional file(s).

## Abbreviations

TMEM106B: Transmembrane protein 106B
FTLD: Frontotemporal lobar degeneration (FTLD),
AD: Alzheimer’s disease
CTE: Chronic traumatic encephalopathy
LATE: Limbic-predominant age-related TDP- 43 encephalopathy
SNP: Single nucleotide polymorphisms
PGRN: Progranulin
MEF: Mouse embryonic fibroblasts
TEM: Transmission Electron Microscopy
RIN: RNA integrity number
DEA: Differential expression analysis
DEG: Differentially expressed genes
MWM: Morris Water Maze
OF: Open field
CF: Contextual Fear
PA: Passive avoidance
EPM: Elevated Plus Maze
NOR: Novel Object Recognition
FST: Forced Swim test
TST: Tail Suspension Test
AID: Activity-regulated Inhibitor of Death
NTRK: Neurotrophic tyrosine receptor kinase
TRK: Tropomyosin receptor kinase
MAPK: Mitogen-activated protein kinases
CREB: CAMP response element binding protein
IEG: Immediate–early genes
LTP: Long-term Potentiation
PTP: Post-tetanic Potentiation
PPR: Paired-Pulse Ratio
CTF: C-terminal fragment
