# Supplementary figures and images for: Increased TMEM106B levels lead to lysosomal dysfunction which affects synaptic signaling and neuronal health
Source: Mol Neurodegener. 2025 Apr 23;20:45. doi: 10.1186/s13024-025-00831-2 (PMC12016085; doi:10.1186/s13024-025-00831-2)

Figure S1.

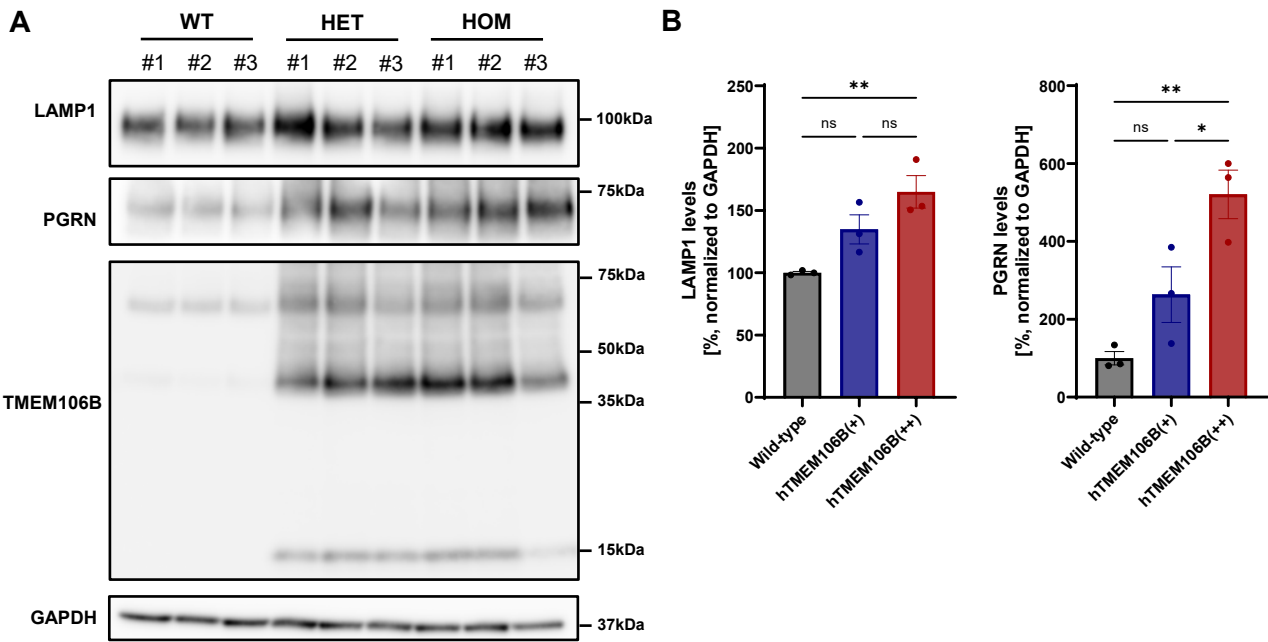

**Figure S2.**

**Wild-type**

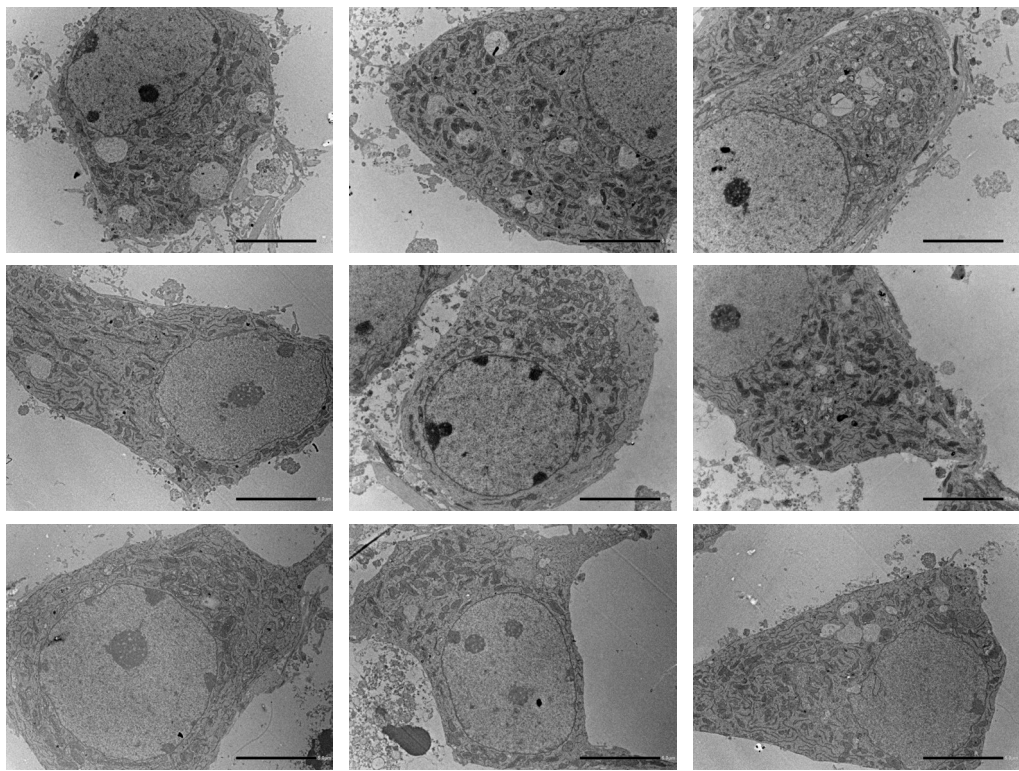

**hTMMEM106B(+)**

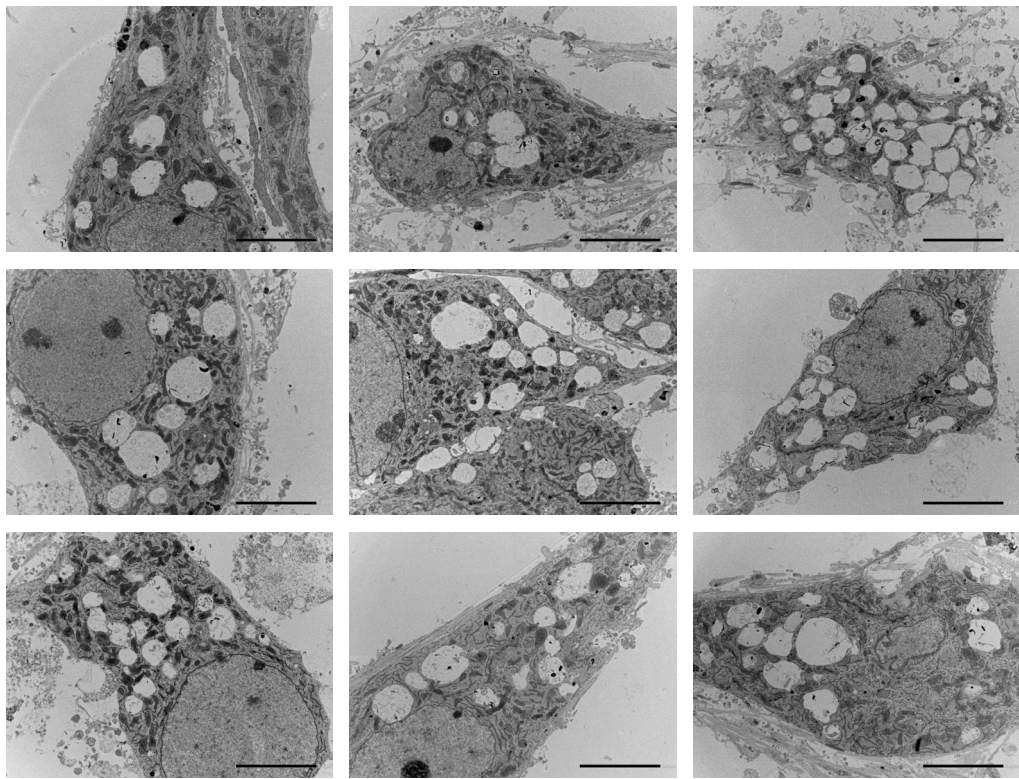

**Figure S3.**

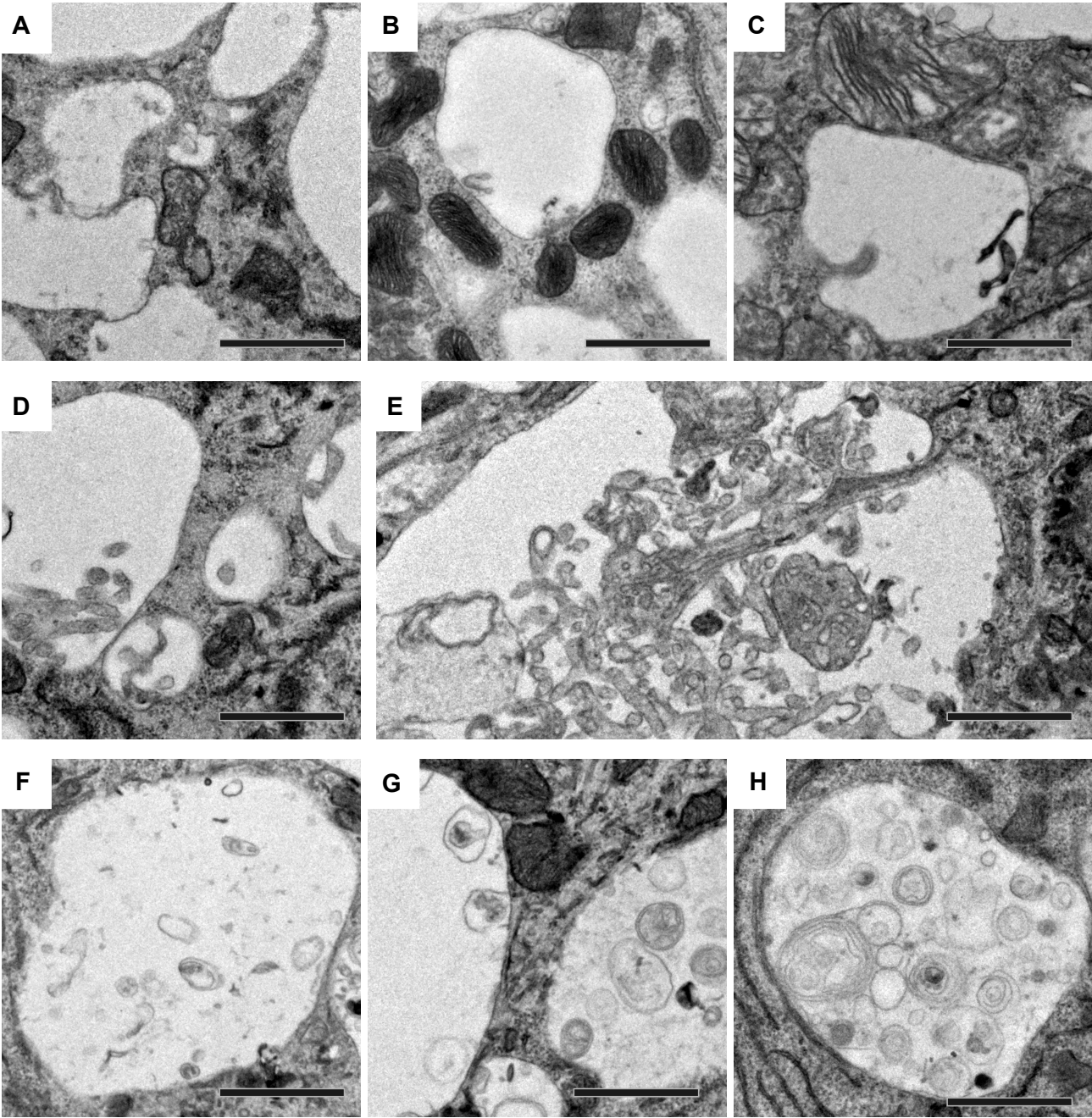

Figure S4.

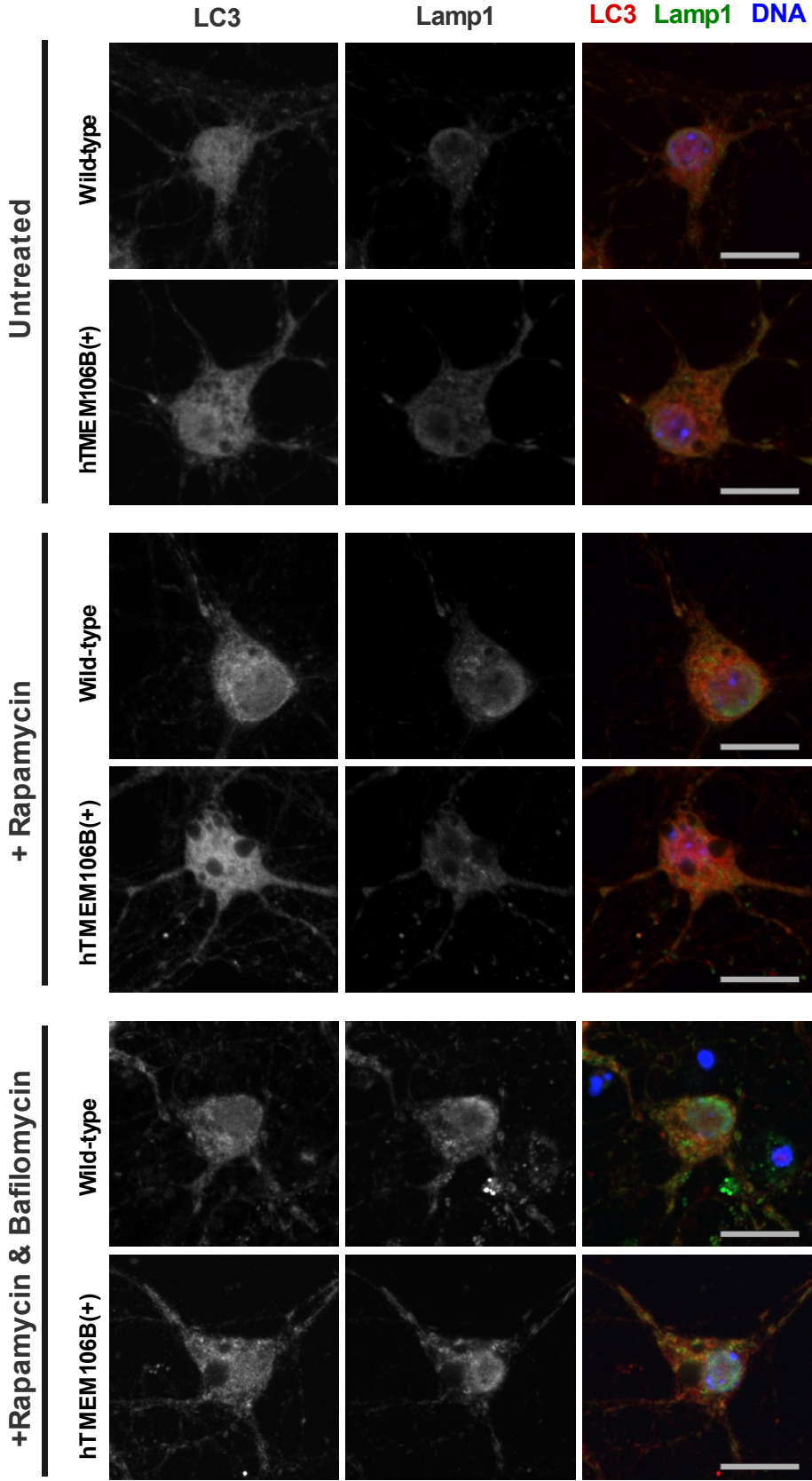

**Figure S5.**

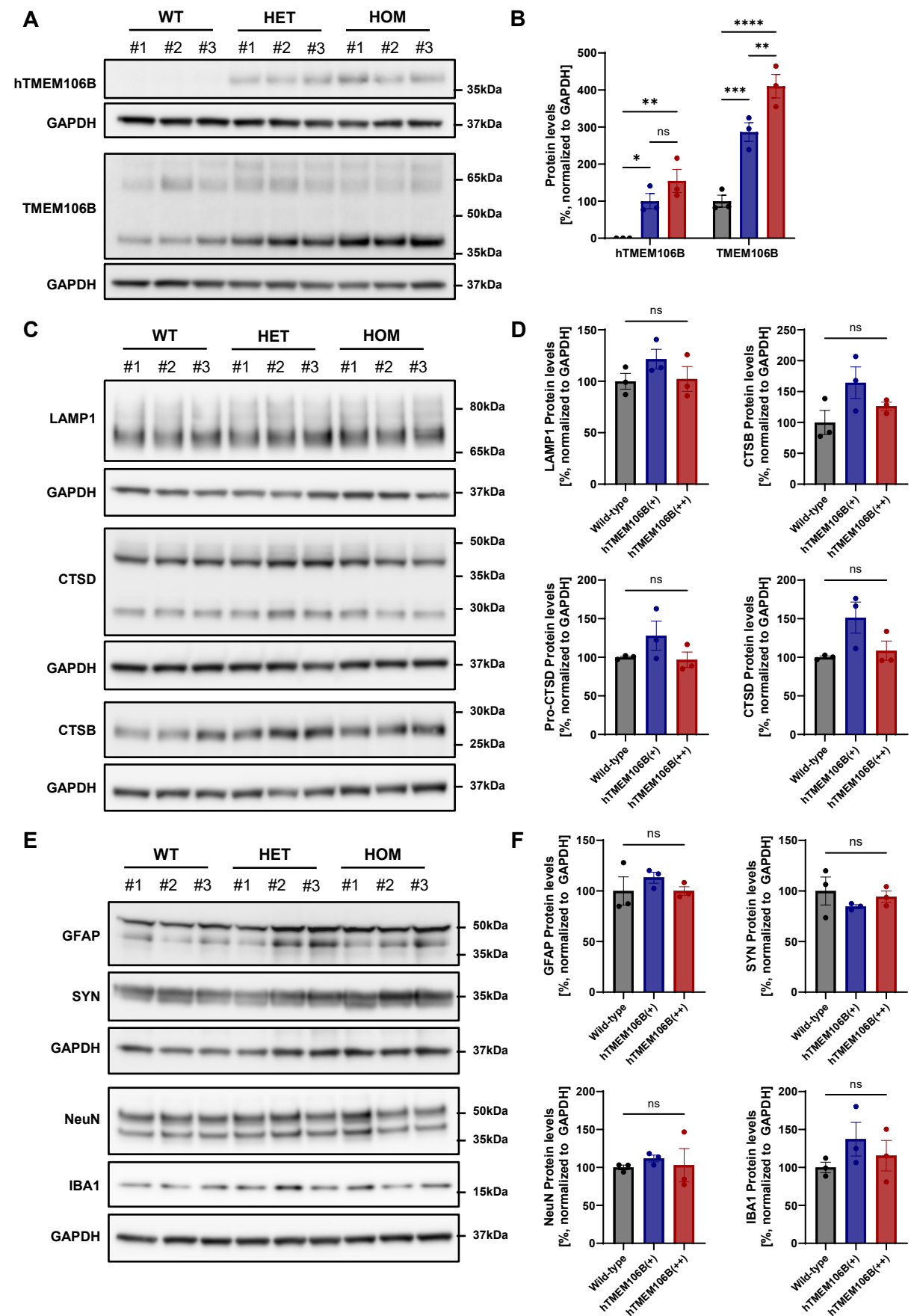

Figure S6.

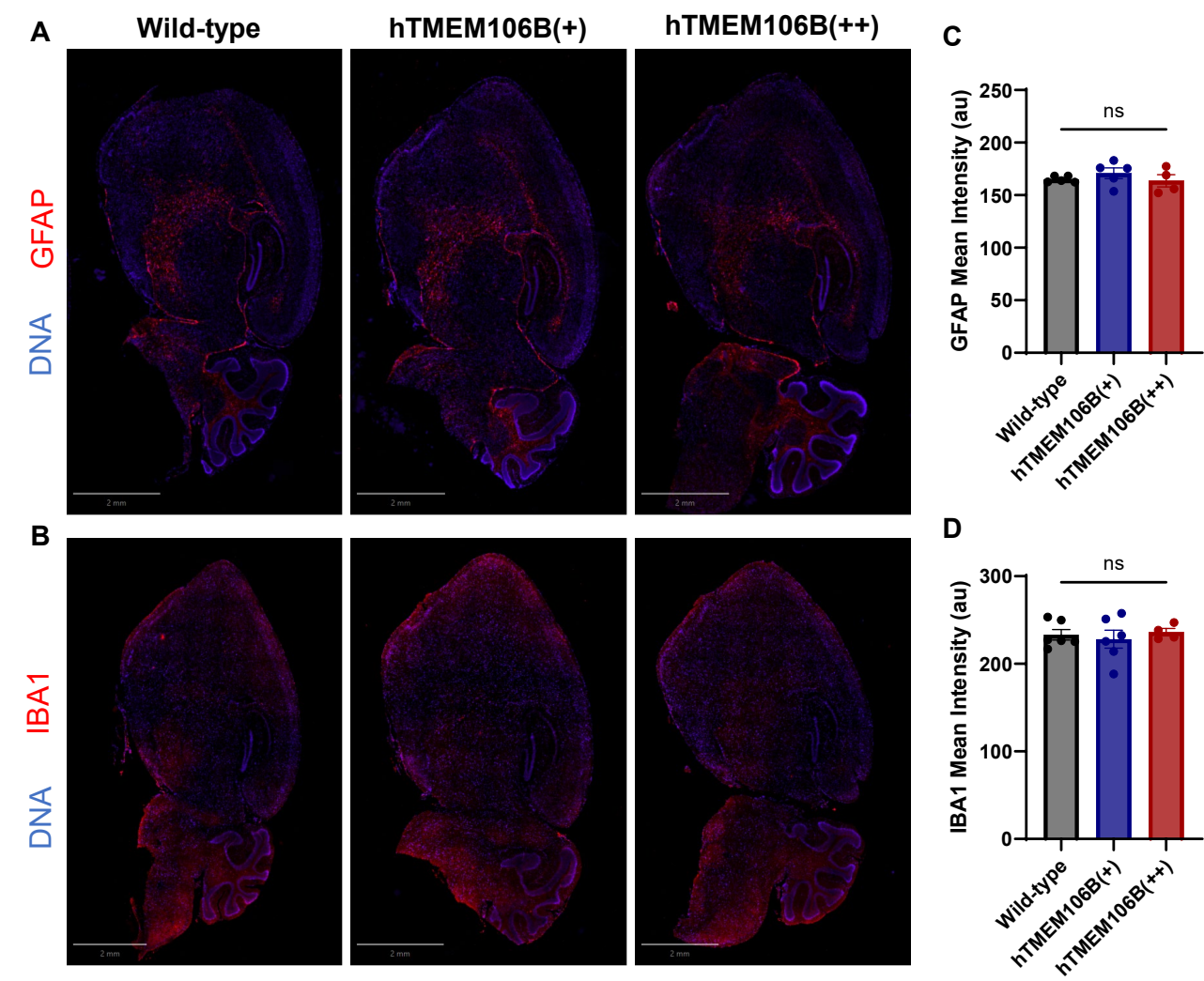

Figure S7.

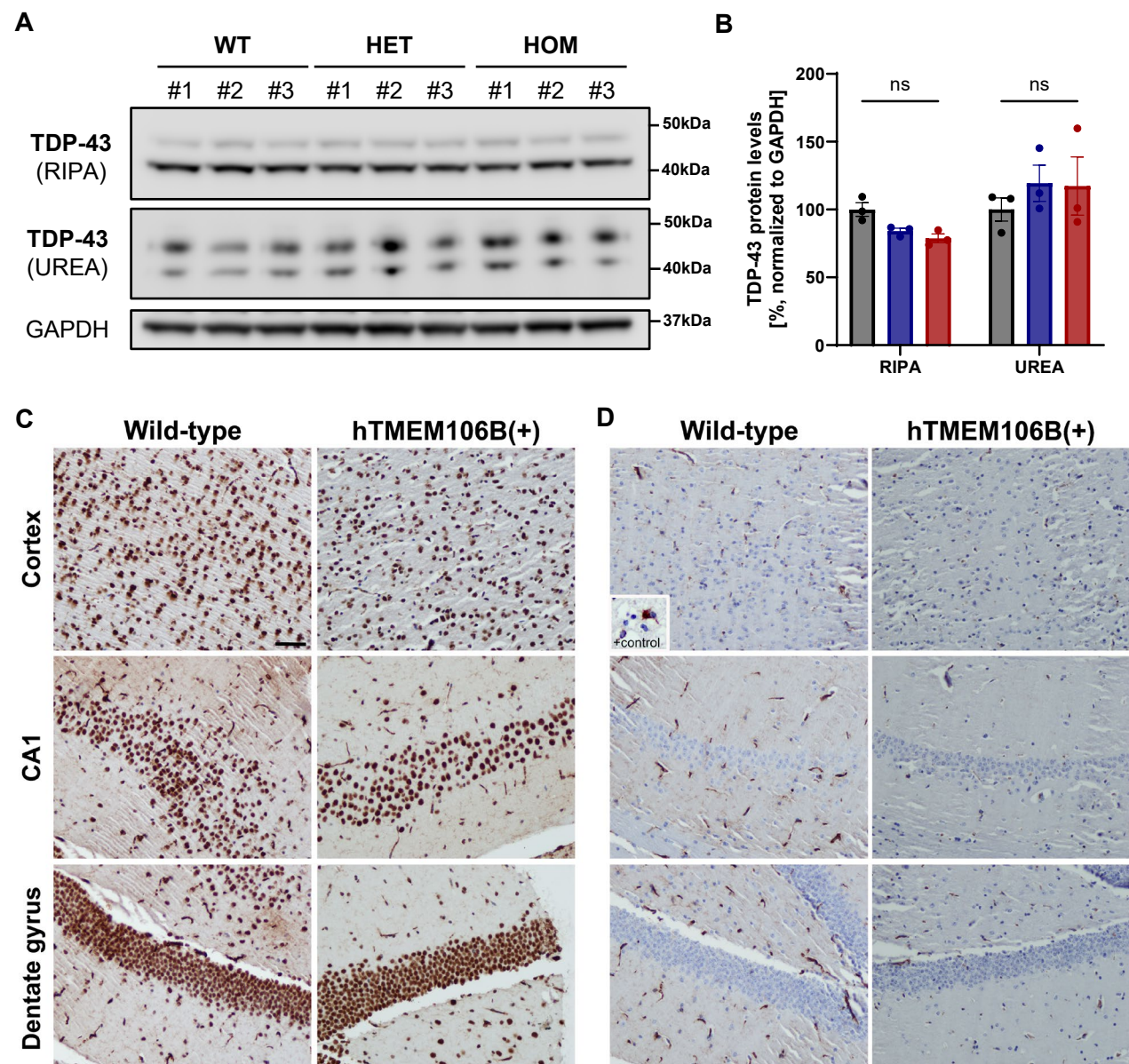

**Figure S8.**

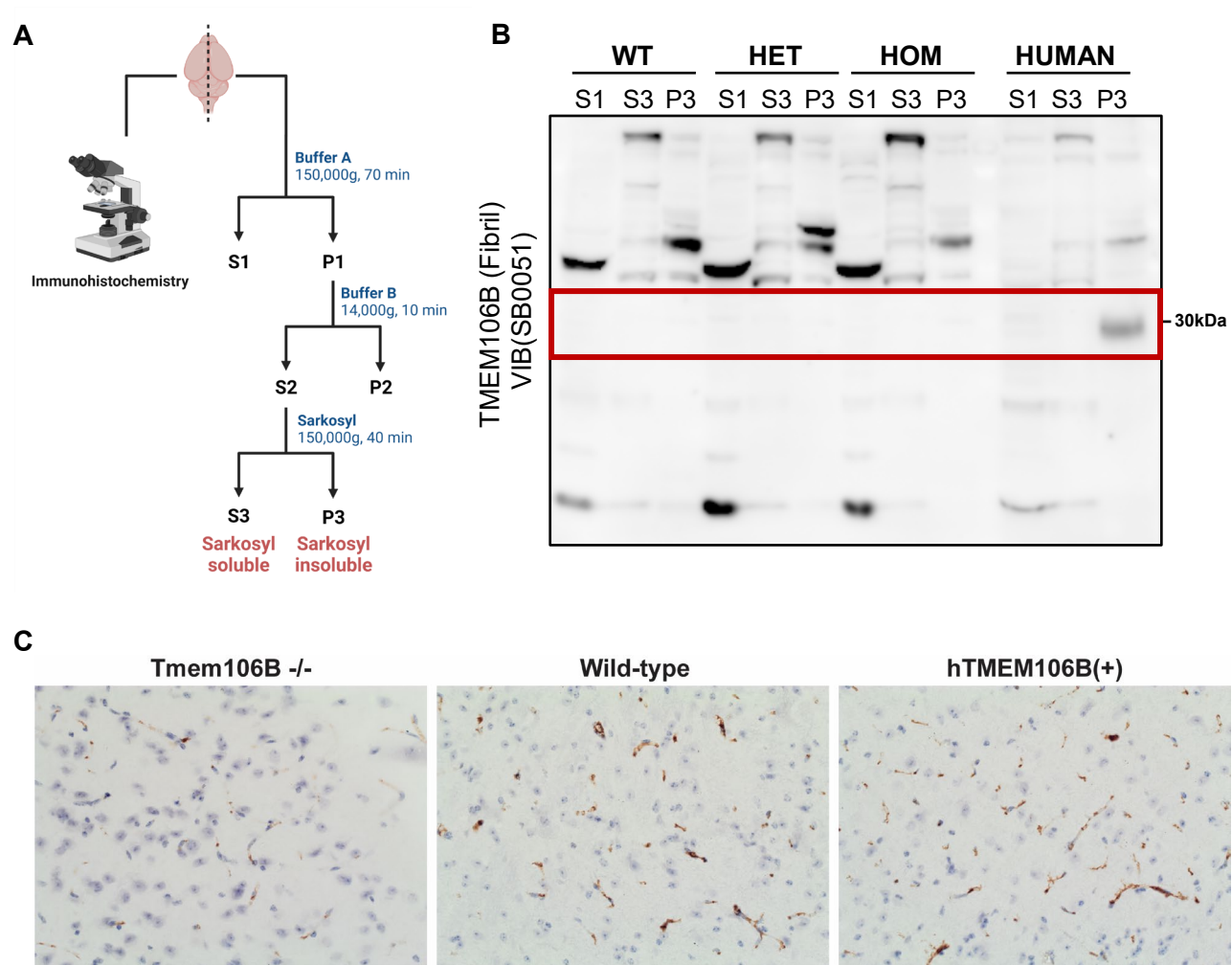

Supplement: Supplementary file 1 — Additional file 1: Figure S1. TMEM106B overexpression leads to increased LAMP1 and PGRN levels in MEFs. (A) Immunoblot and (B) quantification of LAMP1, PGRN, and TMEM106B protein levels indicate elevated levels of LAMP1 and PGRN in MEFs derived from TMEM106B overexpression mice which confirms that increased levels of TMEM106B induces lysosomal dysfunction. Data represented as mean ± SEM. One-way ANOVA (n=3/genotype). *, P < 0,05; **, P < 0,01. The 15kDa band represents an N-terminal fragment of TMEM106B of unknown significance which appears to be human-specific but is otherwise uncharacterized. Figure S2. TEM images of wild-type and TMEM106B(+) neurons showing large cytoplasmic vacuoles. Ultrastructural examination with TEM confirmed the presence of numerous cytoplasmic vacuoles with variable content, sizes and shapes in hTMEM106B(+) neurons. While in few wild-type neurons similar structures could be observed, these were far less abundant and generally much smaller in size. Scale bars (5 µm). Figure S3. Ultrastructural characterization of aberrant vacuoles in TMEM106B overexpressing neurons. The electron-lucent cytoplasmatic vacuoles are enclosed by single membranes. Many vacuoles are largely empty, or contain only small irregularly shaped cytosolic components A,B,C). Cytoplasmic invaginations can penetrate the vacuoles (C,D), in rare cases resembling a network of cytoplasm stretches (E). Some vacuoles contain mostly degraded material (F), while others mostly hold a build-up of membranes and vesicles (G, H). Scale bars (1µm). Figure S4. The vacuoles do not colocalize with the autophagosome marker LC3. Representative images of wild-type and hTMEM106B(+) stained for LAMP1 and LC3. Neurons were treated with rapamycin (500nM) and/or bafilomycin (50nM) for 4 h to induce autophagosome formation. The majority of the vacuoles do not colocalize with LC3. Only few vacuoles were associated with autophagosomes. Scale bars (20µm). Figure S5. TMEM106B overexpression does not lead [file 13024_2025_831_MOESM1_ESM.pdf]
