# Supplementary material for: Increased TMEM106B levels lead to lysosomal dysfunction which affects synaptic signaling and neuronal health
Source: Mol Neurodegener. 2025 Apr 23;20:45. doi: 10.1186/s13024-025-00831-2 (PMC12016085; doi:10.1186/s13024-025-00831-2)

UNCROPPED WESTERN BLOTS

Figure 1B

hTMEM106B

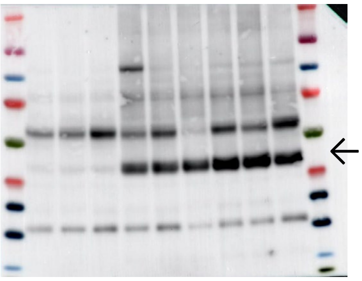

eGFP

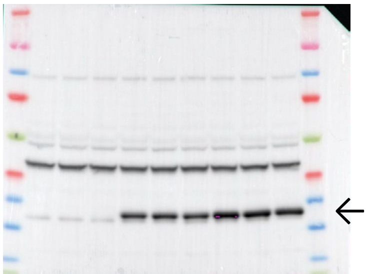

GAPDH

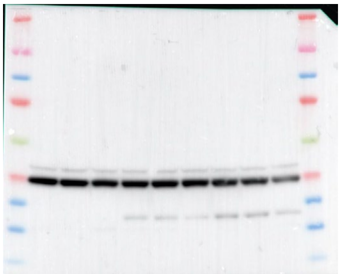

TMEM106B

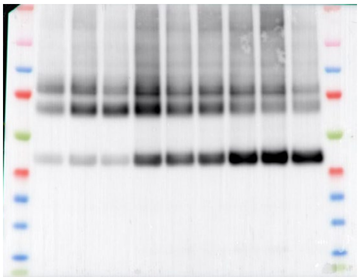

GAPDH

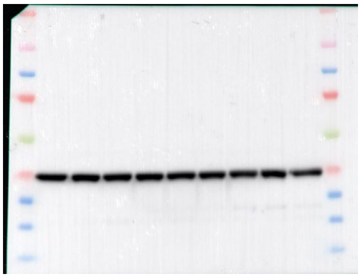

Figure S1

PGRN

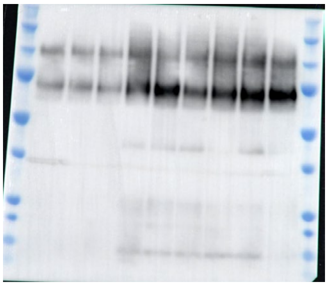

LAMP1

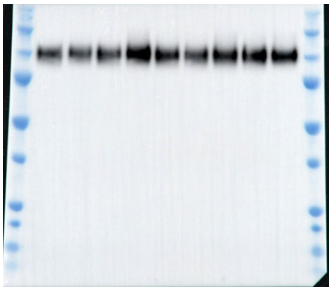

TMEM106B

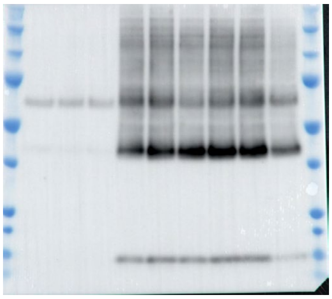

GAPDH

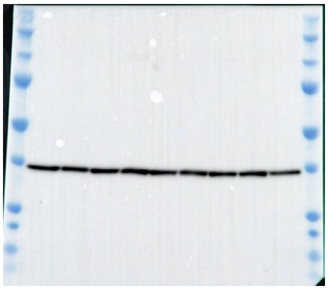

Figure S3A

TMEM106B

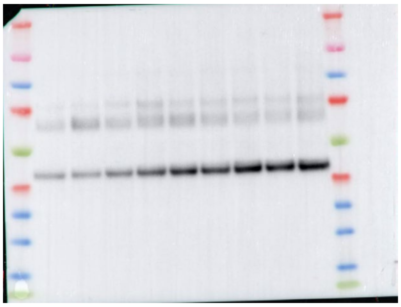

GAPDH

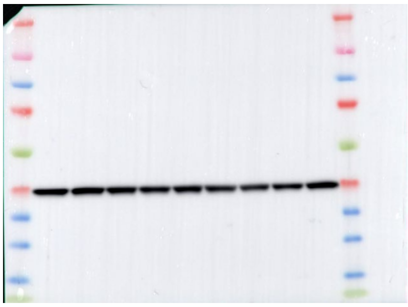

hTMEM106B

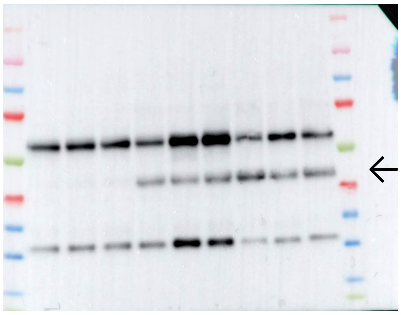

GAPDH

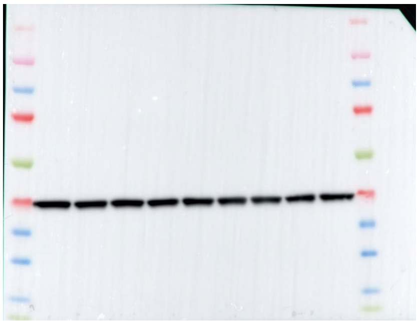

Figure S3C

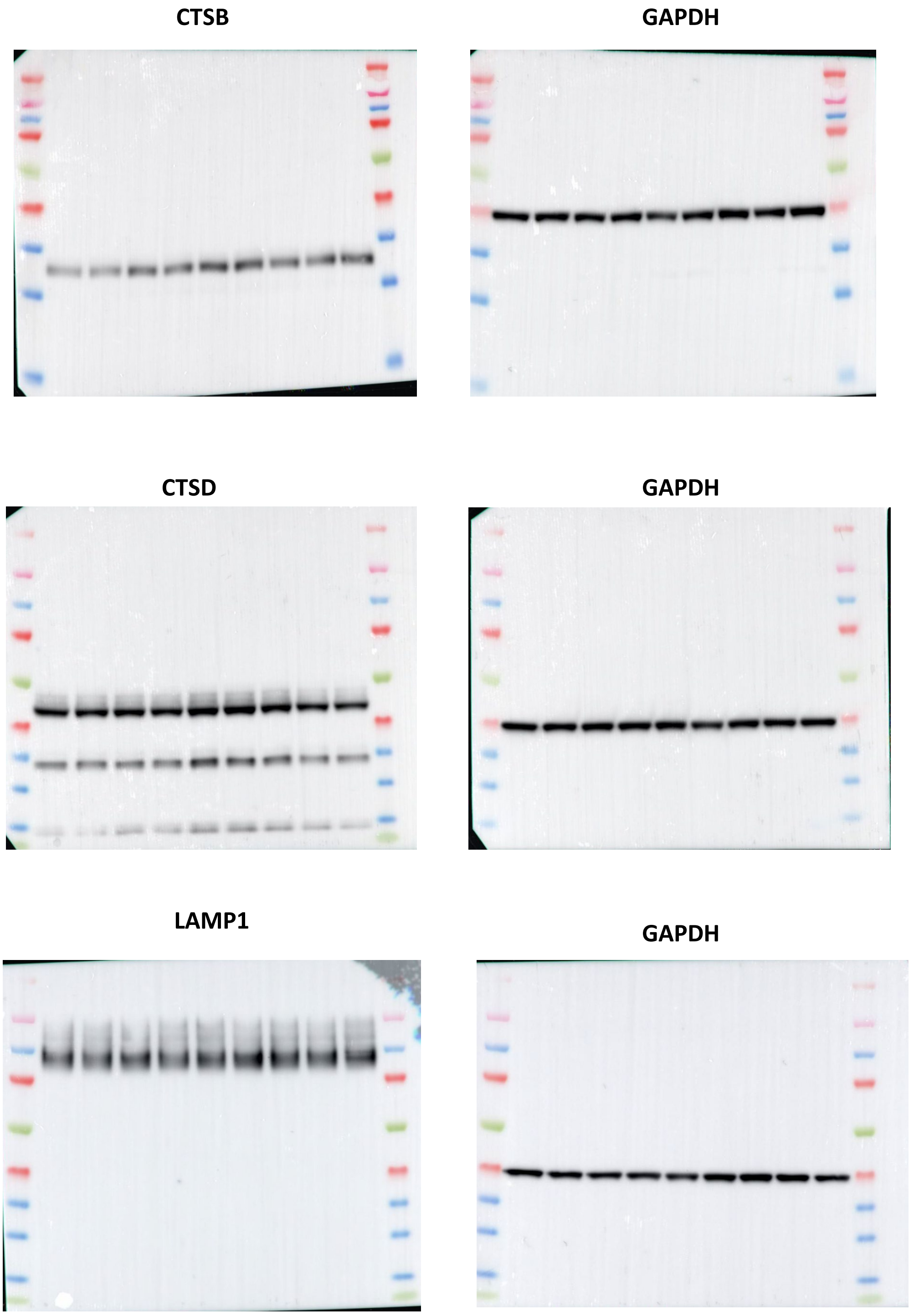

Figure S3E

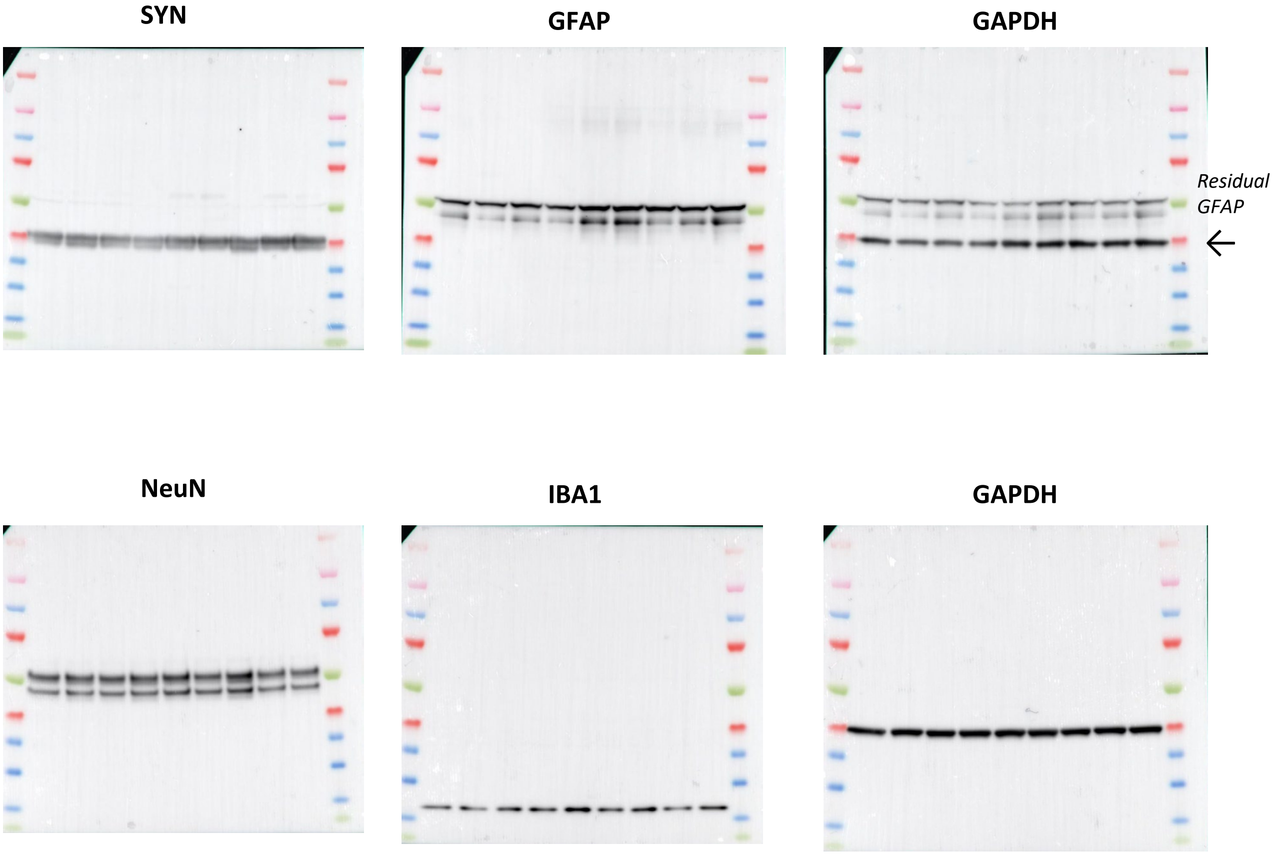

Figure S5A

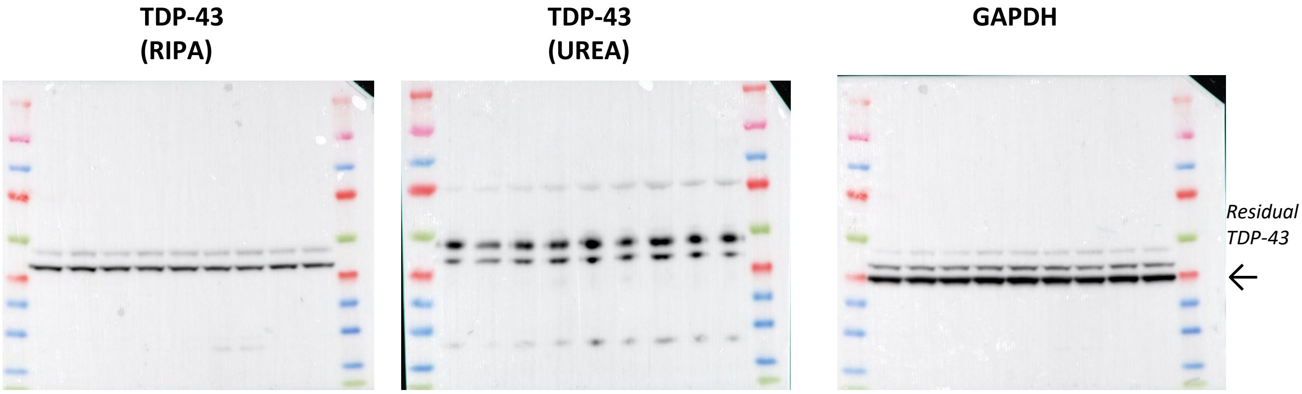

Supplement: Supplementary file 4 — Additional file 4 [file 13024_2025_831_MOESM4_ESM.pdf]
